# Differential expression analysis for sequence count data

**DOI:** 10.1186/gb-2010-11-10-r106

**Published:** 2010-10-27

**Authors:** Simon Anders, Wolfgang Huber

**Affiliations:** 1 European Molecular Biology Laboratory, Mayerhofstraße 1, 69117 Heidelberg, Germany

## Abstract

High-throughput sequencing assays such as RNA-Seq, ChIP-Seq or barcode counting provide quantitative readouts in the form of count data. To infer differential signal in such data correctly and with good statistical power, estimation of data variability throughout the dynamic range and a suitable error model are required. We propose a method based on the negative binomial distribution, with variance and mean linked by local regression and present an implementation, *DESeq*, as an R/Bioconductor package.

## Background

High-throughput sequencing of DNA fragments is used in a range of quantitative assays. A common feature between these assays is that they sequence large amounts of DNA fragments that reflect, for example, a biological system's repertoire of RNA molecules (RNA-Seq [1,2]) or the DNA or RNA interaction regions of nucleotide binding molecules (ChIP-Seq [3], HITS-CLIP [4]). Typically, these reads are assigned to a class based on their mapping to a common region of the target genome, where each class represents a target transcript, in the case of RNA-Seq, or a binding region, in the case of ChIP-Seq. An important summary statistic is the number of reads in a class; for RNA-Seq, this *read count *has been found to be (to good approximation) linearly related to the abundance of the target transcript [2]. Interest lies in comparing read counts between different biological conditions. In the simplest case, the comparison is done separately, class by class. We will use the term *gene *synonymously to class, even though a class may also refer to, for example, a transcription factor binding site, or even a barcode [5].

We would like to use statistical testing to decide whether, for a given gene, an observed difference in read counts is significant, that is, whether it is greater than what would be expected just due to natural random variation.

If reads were independently sampled from a population with given, fixed fractions of genes, the read counts would follow a multinomial distribution, which can be approximated by the Poisson distribution.

Consequently, the Poisson distribution has been used to test for differential expression [6,7]. The Poisson distribution has a single parameter, which is uniquely determined by its mean; its variance and all other properties follow from it; in particular, the variance is equal to the mean. However, it has been noted [1,8] that the assumption of Poisson distribution is too restrictive: it predicts smaller variations than what is seen in the data. Therefore, the resulting statistical test does not control type-I error (the probability of false discoveries) as advertised. We show instances for this later, in the Discussion.

To address this so-called overdispersion problem, it has been proposed to model count data with negative binomial (NB) distributions [9], and this approach is used in the *edgeR *package for analysis of SAGE and RNA-Seq [8,10]. The NB distribution has parameters, which are uniquely determined by mean *μ *and variance *σ*^2^. However, the number of replicates in data sets of interest is often too small to estimate both parameters, mean and variance, reliably for each gene. For *edgeR*, Robinson and Smyth assumed [11] that mean and variance are related by *σ*^2 ^= *μ *+ *αμ*^2^, with a single proportionality constant *α *that is the same throughout the experiment and that can be estimated from the data. Hence, only one parameter needs to be estimated for each gene, allowing application to experiments with small numbers of replicates.

In this paper, we extend this model by allowing more general, data-driven relationships of variance and mean, provide an effective algorithm for fitting the model to data, and show that it provides better fits (Section *Model*). As a result, more balanced selection of differentially expressed genes throughout the dynamic range of the data can be obtained (Section *Testing for differential expression*). We demonstrate the method by applying it to four data sets (Section *Applications*) and discuss how it compares to alternative approaches (Section *Conclusions*).

## Results and Discussion

### Model

#### Description

We assume that the number of reads in sample *j *that are assigned to gene *i *can be modeled by a negative binomial (NB) distribution,

(1) $$K_{ij}\sim \text{NB}(\mu_{ij},\sigma_{ij}^{2}),$$

which has two parameters, the mean *μ_ij _*and the variance $\sigma_{ij}^{2}$. The read counts *K_ij _*are non-negative integers. The probabilities of the distribution are given in Supplementary Note A. (All Supplementary Notes are in Additional file 1.) The NB distribution is commonly used to model count data when overdispersion is present [12].

In practice, we do not know the parameters *μ_ij _*and $\sigma_{ij}^{2}$, and we need to estimate them from the data. Typically, the number of replicates is small, and further modelling assumptions need to be made in order to obtain useful estimates. In this paper, we develop a method that is based on the following three assumptions.

First, the mean parameter *μ_ij_*, that is, the expectation value of the observed counts for gene *i *in sample *j*, is the product of a condition-dependent per-gene value *q*_*i*, *ρ*(*j*) _(where *ρ*(*j*) is the experimental condition of sample *j*) and a size factor *s*_*j*_,

(2) $$\mu_{ij}=q_{i,\rho {\left(j\right)}^{S}j}.$$

*q*_*i,ρ*(*j*) _is proportional to the expectation value of the true (but unknown) concentration of fragments from gene *i *under condition *ρ*(*j*). The size factor *s*_*j *_represents the coverage, or sampling depth, of library *j*, and we will use the term *common scale *for quantities, such as *q*_*i*, *ρ*(*j*)_, that are adjusted for coverage by dividing by *s*_*j*_.

Second, the variance $\sigma_{ij}^{2}$ is the sum of a *shot noise term *and a *raw variance term*,

(3) $$\sigma_{ij}^{2}=\underset{\text{shot}\;\;\text{noise}}{\underset{︸}{\mu_{ij}}}+\underset{\text{raw}\;\;\text{variance}}{\underset{︸}{s_{j}^{2}v_{i,\rho (j)}}}.$$

Third, we assume that the per-gene raw variance parameter *v*_*i*, *ρ *_is a smooth function of *q*_*i*_, *ρ*,

(4) $$v_{i,\rho (j)}=v_{\rho }(q_{i,\rho (j)}).$$

This assumption is needed because the number of replicates is typically too low to get a precise estimate of the variance for gene *i *from just the data available for this gene. This assumption allows us to pool the data from genes with similar expression strength for the purpose of variance estimation.

The decomposition of the variance in Equation (3) is motivated by the following hierarchical model: We assume that the actual concentration of fragments from gene *i *in sample *j *is proportional to a random variable *R_ij_*, such that the rate that fragments from gene *i *are sequenced is *s_j_r_ij_*. For each gene *i *and all samples *j *of condition *ρ*, the *R_ij _*are i.i.d. with mean *q_iρ _*and variance *v_iρ_*. Thus, the count value *K_ij_*, conditioned on *R_ij _*= *r_ij_*, is Poisson distributed with rate *s_j_r_ij_*. The marginal distribution of *K_ij _*- when allowing for variation in *R_ij _*- has the mean *μ_ij _*and (according to the law of total variance) the variance given in Equation (3). Furthermore, if the higher moments of *R_ij _*are modeled according to a gamma distribution, the marginal distribution of *K_ij _*is NB (see, for example, [12], Section 4.2.2).

#### Fitting

We now describe how the model can be fitted to data. The data are an *n *× *m *table of counts, *k_ij_*, where *i *= 1,..., *n *indexes the genes, and *j *= 1,..., *m *indexes the samples. The model has three sets of parameters:

(i) *m *size factors *s_j_*; the expectation values of all counts from sample *j *are proportional to *s_j_*.

(ii) for each experimental condition *ρ*, *n *expression strength parameters *q_iρ_*; they reflect the expected abundance of fragments from gene *i *under condition *ρ*, that is, expectation values of counts for gene *i *are proportional to *q_iρ_*.

(iii) The smooth functions *v_ρ _*: ℝ^+ ^→ ℝ^+^; for each condition *ρ*, *v_ρ _*models the dependence of the raw variance *v_iρ _*on the expected mean *q_iρ_*.

The purpose of the size factors *s_j _*is to render counts from different samples, which may have been sequenced to different depths, comparable. Hence, the ratios (E*K_ij_*)/(E*K_ij' _*) of expected counts for the same gene *i *in different samples *j *and *j' *should be equal to the size ratio *s_j_*/*s_j' _*if gene *i *is not differentially expressed or samples *j *and *j' *are replicates. The total number of reads, Σ*_i _k_ij_*, may seem to be a good measure of sequencing depth and hence a reasonable choice for *s_j_*. Experience with real data, however, shows this not always to be the case, because a few highly and differentially expressed genes may have strong influence on the total read count, causing the ratio of total read counts not to be a good estimate for the ratio of expected counts.

Hence, to estimate the size factors, we take the median of the ratios of observed counts. Generalizing the procedure just outlined to the case of more than two samples, we use:

(5) $${\hat{s}}_{j}=\underset{i}{\text{median}}\frac{k_{ij}}{{\left(\prod_{v=1}^{m}k_{iv}\right)}^{1/m}}.$$

The denominator of this expression can be interpreted as a pseudo-reference sample obtained by taking the geometric mean across samples. Thus, each size factor estimate ${\hat{s}}_{j}$ is computed as the median of the ratios of the *j*-th sample's counts to those of the pseudo-reference. (Note: While this manuscript was under review, Robinson and Oshlack [13] suggested a similar method.)

To estimate *q_iρ_*, we use the average of the counts from the samples *j *corresponding to condition *ρ*, transformed to the common scale:

(6) $${\hat{q}}_{i\rho }=\frac{1}{m_{\rho }}\sum_{j:\rho (j)=\rho }\frac{k_{ij}}{{\hat{s}}_{j}},$$

where *m_ρ _*is the number of replicates of condition *ρ *and the sum runs over these replicates. the functions *v_ρ_*, we first calculate sample variances on the common scale

(7) $$w_{i\rho }=\frac{1}{m_{\rho }-1}\sum_{j:\rho (j)=\rho }{\left(\frac{k_{ij}}{{\hat{s}}_{j}}-{\hat{q}}_{i\rho }\right)}^{2}$$

and define

(8) $$z_{i\rho }=\frac{{\hat{q}}_{i\rho }}{m_{\rho }}\sum_{j:\rho (j)=\rho }\frac{1}{{\hat{s}}_{j}}.$$

In Supplementary Note B in Additional file 1 we show that *w_iρ _*- *z_iρ _*is an unbiased estimator for the raw variance parameter *v_iρ _*of Equation (3).

However, for small numbers of replicates, *m_ρ_*, as is typically the case in applications, the values *w_iρ _*are highly variable, and *w_iρ _*- *z_iρ _*would not be a useful variance estimator for statistical inference. Instead, we use local regression [14] on the graph $\left({\hat{q}}_{i\rho },w_{i\rho }\right)$ to obtain a smooth function *w_ρ_*(*q*), with

(9) $${\hat{v}}_{\rho }({\hat{q}}_{i\rho })=w_{\rho }({\hat{q}}_{i\rho })-z_{i\rho }$$

as our estimate for the raw variance.

Some attention is needed to avoid estimation biases in the local regression. *w_iρ _*is a sum of squared random variables, and the residuals $w_{i\rho }-w({\hat{q}}_{i\rho })$ are skewed. Following References [15], Chapter 8 and [14], Section 9.1.2, we use a generalized linear model of the gamma family for the local regression, using the implementation in the *locfit *package [16].

### Testing for differential expression

Suppose that we have *m_A _*replicate samples for biological condition A and *m_B _*samples for condition B. For each gene *i*, we would like to weigh the evidence in the data for differential expression of that gene between the two conditions. In particular, we would like to test the null hypothesis *q_iA _*= *q_iB_*, where *q_iA _*is the expression strength parameter for the samples of condition A, and q_iB _for condition B. To this end, we define, as test statistic, the total counts in each condition,

(10) $$\begin{array}{cc} K_{i\text{A}}=\sum_{j:\rho (j)=\text{A}}K_{ij}, & K_{i\text{B}}=\sum_{j:\rho (j)=\text{B}}K_{ij}, \end{array}$$

and their overall sum *K_iS _*= *K_iA _*+ *K_iB_*. From the error model described in the previous Section, we show below that - under the null hypothesis - we can compute the probabilities of the events *K_iA _*= *a *and *K_iB _*= *b *for any pair of numbers *a *and *b*. We denote this probability by *p*(*a*, *b*). The *P *value of a pair of observed count sums (*k_iA_*, *k_iB_*) is then the sum of all probabilities less or equal to *p*(*k_iA_*, *k_iB_*), given that the overall sum is *k_iS_*:

(11) $$p_{i}=\frac{\sum_{\begin{array}{c} a+b=k_{i\text{S}}\\ p(a,b)\leq p(k_{i\text{A}}k_{i\text{B}}) \end{array}}p(a,b)}{\sum_{a+b=k_{i\text{S}}}p(a,b)}.$$

The variables *a *and *b *in the above sums take the values 0,..., *k*_*i*S_. The approach presented so far follows that of Robinson and Smyth [11] and is analogous to that taken by other conditioned tests, such as Fisher's exact test. (See Reference [17], Chapter 3 for a discussion of the merits of conditioning in tests.)

**Computation of ***p*(*a*, *b*). First, assume that, under the null hypothesis, counts from different samples are independent. Then, *p*(*a*, *b*) = Pr(*K*_*i*A _= *a*) Pr(*K*_*i*B _= *b*). The problem thus is computing the probability of the event *K*_*i*A _= *a*, and, analogously, of *K*_*i*B _= *b*. The random variable *K*_*i*A _is the sum of *m*_*A*_

NB-distributed random variables. We approximate its distribution by a NB distribution whose parameters we obtain from those of the *K*_*ij*_. To this end, we first compute the pooled mean estimate from the counts of both conditions,

(12) $${\hat{q}}_{i0}=\sum_{j:\rho (j)\in \{A,B\}}k_{ij}/s_{j},$$

which accounts for the fact that the null hypothesis stipulates that *q*_*i*A _= *q*_*i*B_. The summed mean and variance for condition A are

(13) $${\hat{\mu }}_{i\text{A}}=\sum_{j\in \text{A}}s_{j}{\hat{q}}_{i0},$$

(14) $${\hat{\sigma }}_{i\text{A}}^{2}=\sum_{j\in \text{A}}{\hat{s}}_{j}{\hat{q}}_{i0}+{\hat{s}}_{j}^{2}{\hat{v}}_{\text{A}}({\hat{q}}_{i0}).$$

Supplementary Note C in Additional file 1 describes how the distribution parameters of the NB for *K*_*i*A _can be determined from ${\hat{\mu }}_{i\text{A}}$ and ${\hat{\sigma }}_{i\text{A}}^{2}$. (To avoid bias, we do not match the moments directly, but instead match a different pair of distribution statistics.) The parameters of *K*_*i*B _are obtained analogously.

Supplementary Note D in Additional file 1 explains how we evaluate the sums in Equation (11).

### Applications

#### Data sets

We present results based on the following data sets:

##### RNA-Seq in fly embryos

B. Wilczynski, Y.-H. Liu, N. Delhomme and E. Furlong have conducted RNA-Seq experiments in fly embryos and kindly shared part of their data with us ahead of publication. In each sample of this data set, a gene was engineered to be over-expressed, and we compare two biological replicates each of two such conditions, in the following denoted as 'A' and 'B'.

##### Tag-Seq of neural stem cells

Engström *et al. *[18] performed Tag-Seq [19] for tissue cultures of neural cells, including four from glioblastoma-derived neural stem-cells ('GNS') and two from non-cancerous neural stem ('NS') cells. As each tissue culture was derived from a different subject and so has a different genotype, these data show high variability.

##### RNA-Seq of yeast

Nagalakshmi *et al. *[1] performed RNA-Seq on replicates of *Saccharomyces cerevisiae *cultures. They tested two library preparation protocols, *dT *and *RH*, and obtained three sequencing runs for each protocol, such that for the first run of each protocol, they had one further technical replicate (same culture, replicated library preparation) and one further biological replicate (different culture).

##### ChIP-Seq of HapMap samples

Kasowski *et al. *[20] compared protein occupation of DNA regions between ten human individuals by ChIP-Seq. They compiled a list of regions for polymerase II and NF-κB, and counted, for each sample, the number of reads that mapped onto each region. The aim of the study was to investigate how much the regions' occupation differed between individuals.

#### Variance estimation

**Figure 1:**
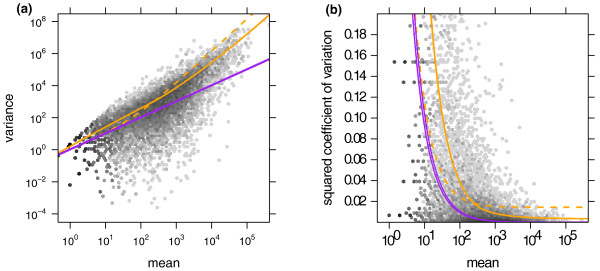
**Dependence of the variance on the mean for condition *A *in the fly RNA-Seq data**. (a) The scatter plot shows the common-scale sample variances (Equation (7)) plotted against the common-scale means (Equation (6)). The orange line is the fit *w*(*q*). The purple lines show the variance implied by the Poisson distribution for each of the two samples, that is, ${\hat{s}}_{j}{\hat{q}}_{i,A}$. The dashed orange line is the variance estimate used by *edgeR*. (b) Same data as in (a), with the *y*-axis rescaled to show the squared coefficient of variation (SCV), that is all quantities are divided by the square of the mean. In (b), the solid orange line incorporated the bias correction described in Supplementary Note C in Additional file 1. (The plot only shows SCV values in the range [0, 0.2]. For a zoom-out to the full range, see Supplementary Figure S9 in Additional file 1.)

We start by demonstrating the variance estimation. Figure 1a shows the sample variances *w*_*iρ *_(Equation (7)) plotted against the means ${\hat{q}}_{i\rho }$ (Equation (6)) for condition *A *in the fly RNA-Seq data. Also shown is the local regression fit *w_ρ_*(*q*) and the shot noise ${\hat{s}}_{j}{\hat{q}}_{i\rho }$. In Figure 1b, we plotted the squared coefficient of variation (SCV), that is the ratio of the variance to the mean squared. In this plot, the distance between the orange and the purple line is the SCV of the noise due to biological sampling (cf. Equation (3)).

The many data points in Figure 1b that lie far above the fitted orange curve may let the fit of the local regression appear poor. However, a strong skew of the residual distribution is to be expected. See Supplementary Note E in Additional file 1 for details and a discussion of diagnostics suitable to verify the fit.

#### Testing

**Figure 2:**
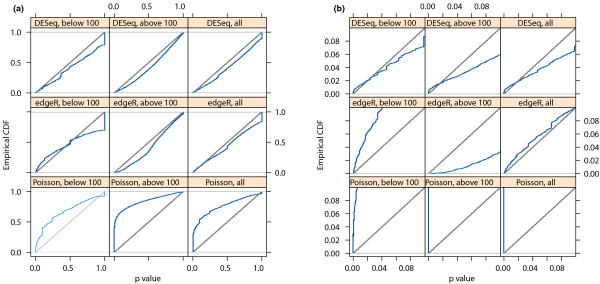
**Type-I error control**. The panels show empirical cumulative distribution functions (ECDFs) for *P *values from a comparison of one replicate from condition *A *of the fly RNA-Seq data with the other one. No genes are truly differentially expressed, and the ECDF curves (blue) should remain below the diagonal (gray). Panel (a): top row corresponds to *DESeq*, middle row to *edgeR *and bottom row to a Poisson-based *χ*^2 ^test. The right column shows the distributions for all genes, the left and middle columns show them separately for genes below and above a mean of 100. Panel (b) shows the same data, but zooms into the range of small *P *values. The plots indicate that *edgeR *and *DESeq *control type I error at (and in fact slightly below) the nominal rate, while the Poisson-based *χ*^2 ^test fails to do so. *edgeR *has an excess of small *P *values for low counts: the blue line lies above the diagonal. This excess is, however, compensated by the method being more conservative for high counts. All methods show a point mass at *p *= 1, this is due to the discreteness of the data, whose effect is particularly evident at low counts.

**Figure 3:**
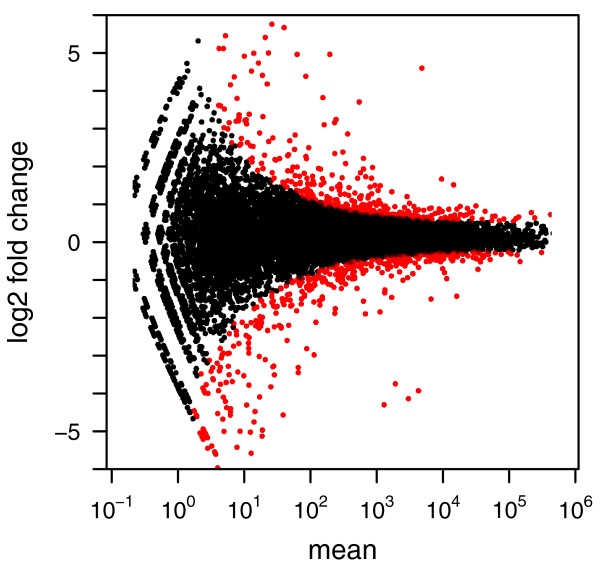
**Testing for differential expression between conditions *A *and *B*: Scatter plot of log_2 _ratio (fold change) versus mean**. The red colour marks genes detected as differentially expressed at 10% false discovery rate when Benjamini-Hochberg multiple testing adjustment is used. The symbols at the upper and lower plot border indicate genes with very large or infinite log fold change. The corresponding volcano plot is shown in Supplementary Figure S8 in Additional file 2.

In order to verify that *DESeq *maintains control of type-I error, we contrasted one of the replicates for condition *A *in the fly data against the other one, using for both samples the variance function estimated from the two replicates. Figure 2 shows the empirical cumulative distribution functions (ECDFs) of the *P *values obtained from this comparison. To control type-I error, the proportion of *P *values below a threshold *α *has to be ≤ *α*, that is, the ECDF curve (blue line) should not get above the diagonal (gray line). As the figure indicates, type-I error is controlled by *edgeR *and *DESeq*, but not by a Poisson-based *χ*^2 ^test. The latter underestimates the variability of the data and would thus make many false positive rejections. In addition to this evaluation on real data, we also verified *DESeq*'s type-I error control on simulated data that were generated from the error model described above; see Supplementary Note G in Additional file 1. Next, we contrasted the two *A *samples against the two *B *samples. Using the procedure described in the previous Section, we computed a *P *value for each gene. Figure 3 shows the obtained fold changes and *P *values. 12% of the P values were below 5%. Adjustment for multiple-testing with the procedure of Benjamini and Hochberg [21] yielded significant differential expression at false discovery rate (FDR) of 10% for 864 genes (of 17,605). These are marked in red in the figure. Figure 3 demonstrates how the ability to detect differential expression depends on overall counts. Specifically, the strong shot noise for low counts causes the testing procedure to call only very high fold changes significant. It can also be seen that, for counts below approximately 100, even a small increase in count levels reduces the impact of shot noise and hence the fold-change requirement, while at higher counts, when shot noise becomes unimportant (cf. Figure 1b), the fold-change cut-off depends only weakly on count level. These plots are helpful to guide experiment design: For weakly expressed genes, in the region where shot noise is important, power can be increased by deeper sequencing, while for the higher-count regime, increased power can only be achieved with further biological replicates.

#### Comparison with edgeR

We also analyzed the data with *edgeR *(version 1.6.0; [8,10,11]). We ran *edgeR *with four different settings, namely in common-dispersion and in tagwise-dispersion mode, and either using the size factors as estimated by *DESeq *or taking the total numbers of sequenced reads. The results did not depend much on these choices, and here we report the results for tag-wise dispersion mode with *DESeq*-estimated size factors. (The R code required to reproduce all analyses, figures and numbers reported in this article is provided in Additional file 2; in addition, this supplement provides the results for the other settings of *edgeR*. The raw data can be found in Additional file 3.)

Going back to Figure 1 we see that *edgeR*'s single-value dispersion estimate of the variance is lower than that of *DESeq *for weakly expressed genes and higher for strongly expressed genes. As a consequence, as we have seen in Figure 2*edgeR *is anti-conservative for lowly expressed genes. However, it compensates for this by being more conservative with strongly expressed genes, so that, on average, type-I error control is maintained.

**Figure 4:**
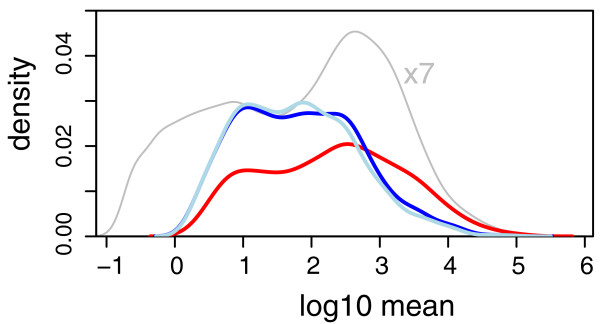
**Distribution of hits through the dynamic range**. The density of common-scale mean values *q_i _*for all genes in the fly data (gray line, scaled down by a factor of seven), and for the hits reported by *DESeq *(red line) and by *edgeR *at a false discovery rate of 10% (dark blue line: with tag-wise dispersion estimation; light blue line: common dispersion mode).

Nevertheless, in a test between different conditions, this behavior can result in a bias in the list of discoveries; for the present data, as Figure 4 shows, weakly expressed genes seem to be overrepresented, while very few genes with high average level are called differentially expressed by *edgeR*. While overall the sensitivity of both methods seemed comparable (*DESeq *reported 864 hits, *edgeR *1, 127 hits), *DESeq *produced results which were more balanced over the dynamic range.

Similar results were obtained with the neural stem cell data, a data set with a different biological background and different noise characteristics (see Supplementary Note F in Additional file 1). The flexibility of the variance estimation scheme presented in this work appears to offer real advantages over the existing methods across a range of applications.

#### Working without replicates

*DESeq *allows analysis of experiments with no biological replicates in one or even both of the conditions. While one may not want to draw strong conclusions from such an analysis, it may still be useful for exploration and hypothesis generation.

If replicates are available only for one of the conditions, one might choose to assume that the variance-mean dependence estimated from the data for that condition holds as well for the unreplicated one.

If neither condition has replicates, one can still perform an analysis based on the assumption that for most genes, there is no true differential expression, and that a valid mean-variance relationship can be estimated from treating the two samples as if they were replicates. A minority of differentially abundant genes will act as outliers; however, they will not have a severe impact on the gamma-family GLM fit, as the gamma distribution for low values of the shape parameter has a heavy right-hand tail. Some overestimation of the variance may be expected, which will make that approach conservative.

We performed such an analysis with the fly RNA-Seq and the neural cell Tag-Seq data, by restricting both data sets to only two samples, one from each condition. For the neural cell data, the estimated variance function was, as expected, somewhat above the two functions estimated from the *GNS *and *NS *replicates.

Using it to test for differential expression still found 269 hits at FDR = 10%, of which 202 were among the 612 hits from the more reliable analysis with all available samples. In the case of the fly RNA-Seq data, however, only 90 of the 862 hits (11%) were recovered (with two new hits). These observations are explained by the fact that in the neural cell data, the variability between replicates was not much smaller than between conditions, making the latter a usable surrogate for the former. On the other hand, for the fly data, the variability between replicates was much smaller than between the conditions, indicating that the replication provided important and otherwise not available information on the experimental variation in the data (see also next Section).

#### Variance-stabilizing transformation

Given a variance-mean dependence, a variance-stabilizing transformation (VST) is a monotonous mapping such that for the transformed values, the variance is (approximately) independent of the mean. Using the variance-mean dependence *w*(*q*) estimated by *DESeq*, a VST is given by

(15) $$\tau (\kappa )=\int^{\kappa }\frac{\text{d}q}{\sqrt{w(q)}}.$$

**Figure 5:**
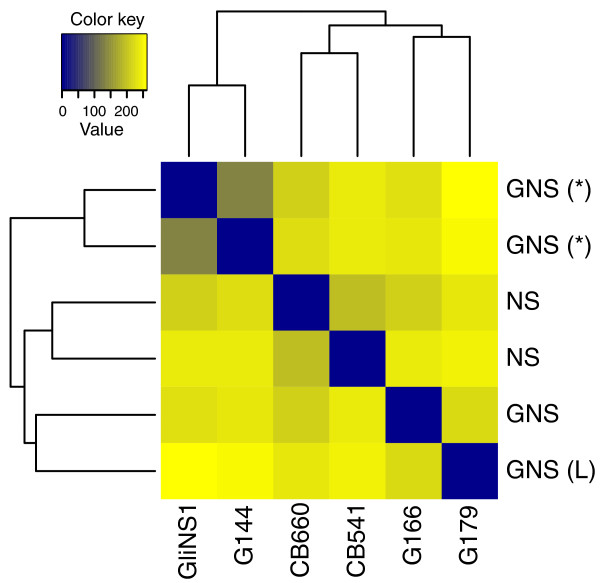
**Sample clustering for the neural cell data of Kasowski et al**. [18]. A common variance function was estimated for all samples and used to apply a variance-stabilizing transformation. The heat map shows a false colour representation of the Euclidean distance matrix (from dark blue for zero distance to orange for large distance), and the dendrogram represents a hierarchical clustering. Two *GNS *samples were derived from the same patient (marked with '(*)') and show the highest degree of similarity. The two other *GNS *samples (including one with atypically large cells, marked '(L)') are as dissimilar from the former as the two *NS *samples.

Applying the transformation *τ *to the common-scale count data, *k_ij_*/*s_j_*, yields values whose variances are approximately the same throughout the dynamic range. One application of VST is sample clustering, as in Figure 5; such an approach is more straightforward than, say, defining a suitable distance metric on the untransformed count data, whose choice is not obvious, and may not be easy to combine with available clustering or classification algorithms (which tend to be designed for variables with similar distributional properties).

#### ChIP-Seq

**Figure 6:**
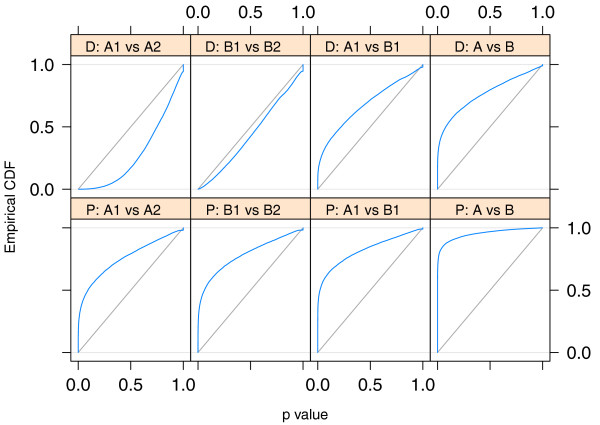
**Application to ChIP-Seq data**. Shown are ECDF curves for *P *values resulting from comparisons of Pol-II ChIP-Seq data between replicates of the same individual (first and second column) and between two different individuals (third and forth column). The upper row corresponds to an analysis with *DESeq *('D'), the lower row to one based on Poisson GLMs ('P'). If no true differential occupation exists (that is, when comparing replicates), the ECDF (blue) should stay below the diagonal (gray), which corresponds to uniform *P *values. In the first column, two replicates from HapMap individual GM12878 (*A1*) were compared against two further replicates from the same individual (*A2*). Similarly, in the second column, two replicates from individual GM12891 (*B1*) were compared against two further replicates from the same individual (*B2*). For *DESeq*, no excess of low *P *values was seen, as expected when comparing replicates. In contrast, the Poisson GLM analysis produced strong enrichments of small *P *values; this is a reflection of overdispersion in the data, that is, the variance in the data was larger than what the Poisson GLM assumes (see also Section *Choice of distribution*). The third column compares two replicates from individual GM12878 (*A1*) against two from the other individual (*B1*). True occupation differences are expected, and both methods result in enrichment of small P values. The forth column shows the comparison of four replicates of GM12878 (*A1 *combined with *A2*) against four replicates of GM12891 (*B1*, *B2*); increased sample size leads to higher detection power and hence smaller *P *values.

*DESeq *can also be used to analyze comparative ChIP-Seq assays. Kasowski *et al. *[20] analyzed transcription factor binding for HapMap individuals and counted for each sample how many reads mapped to pre-determined binding regions. We considered two individuals from their data set, HapMap IDs GM12878 and GM12891, for both of which at least four replicates had been done, and tested for differential occupation of the regions. The upper left two panels of Figure 6 which show comparisons within the same individual, indicate that type-I error was controlled by *DESeq*. No region was significant at 10% FDR using Benjamini-Hochberg adjustment. Differential occupation was found, however, when contrasting the two individuals, with 4,460 of 19,028 regions significant when only two replicates each were used and 8,442 when four replicates were used (upper right two panels).

Using an alternative approach, Kasowski *et al*. fitted generalized linear models (GLMs) of the Poisson family. This (lower row of Figure 6) resulted in an enrichment of small *P *values even for comparisons within the same individual, indicating that the variance was underestimated by the Poisson GLM, and literal use of the P values would lead to anti-conservative (overly optimistic) bias. Kasowski *et al*. addressed this and adjusted for the bias by using additional criteria for calling differential occupation.

## Conclusions

Why is it necessary to develop new statistical methodology for sequence count data? If large numbers of replicates were available, questions of data distribution could be avoided by using non-parametric methods, such as rank-based or permutation tests. However, it is desirable (and possible) to consider experiments with smaller numbers of replicates per condition. In order to compare an observed difference with an expected random variation, we can improve our picture of the latter in two ways: first, we can use distribution families, such as normal, Poisson and negative binomial distributions, in order to determine the higher moments, and hence the tail behavior, of statistics for differential expression, based on observed low order moments such as mean and variance. Second, we can share information, for instance, distributional parameters, between genes, based on the notion that data from different genes follow similar patterns of variability. Here, we have described an instance of such an approach, and we will now discuss the choices we have made.

### Choice of distribution

While for large counts, normal distributions might provide a good approximation of between-replicate variability, this is not the case for lower count values, whose discreteness and skewness mean that probability estimates computed from a normal approximation would be inadequate.

For the Poisson approximation, a key paper is the work by Marioni *et al. *[6], who studied the *technical *reproducibility of RNA-Seq. They extracted total RNA from two tissue samples, one from the liver and one from the kidneys of the same individual. From each RNA sample they took seven aliquots, prepared a library from each aliquot according to the protocol recommended by Illumina and sampled each library on one lane of a Solexa genome analyzer. For each gene, they then calculated the variance of the seven counts from the same tissue sample and found very good agreement with the variance predicted by a Poisson model. In line with our arguments in Section *Model*, Poisson shot noise is the minimum amount of variation to expect in a counting process. Thus, Marioni *et al*. concluded that the technical reproducibility of RNA-Seq is excellent, and that the variation between technical replicates is close to the shot noise limit. From this vantage point, Marioni *et al*. (and similarly Bullard *et al. *[22]) suggested to use the Poisson model (and Fisher's exact test, or a likelihood ratio test as an approximation to it) to test whether a gene is differentially expressed between their two samples. It is important to note that a rejection from such a test only informs us that the difference between the average counts in the two samples is larger than one would expect between *technical *replicates. Hence, we do not know whether this difference is due to the different tissue type, kidney instead of liver, or whether a difference of the same magnitude could have been found as well if one had compared two samples from different parts of the same liver, or from livers of two individuals.

Figure 1 shows that shot noise is only dominant for very low count values, while already for moderate counts, the effect of the biological variation between samples exceeds the shot noise by orders of magnitude.

**Figure 7:**
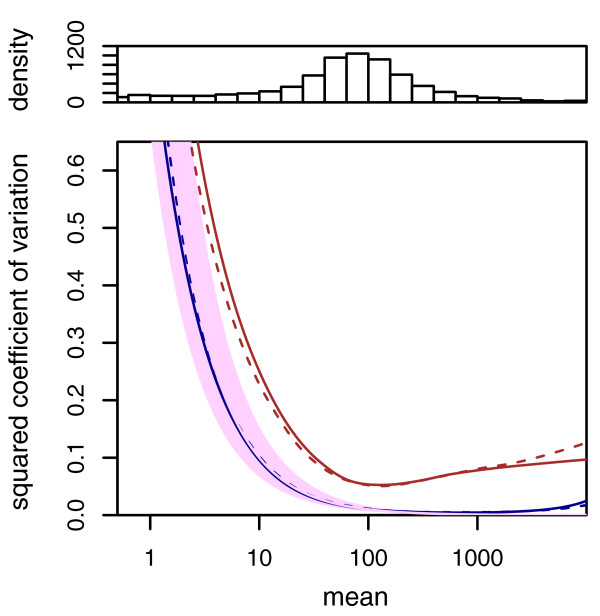
**Noise estimates for the data of Nagalakshmi *et al***. [1]. The data allow assessment of technical variability (between library preparations from aliquots of the same yeast culture) and biological variability (between two independently grown cultures). The blue curves depict the squared coefficient of variation at the common scale, *w_ρ_*(*q*)/*q*^2 ^(see Equation (9)) for technical replicates, the red curves for biological replicates (solid lines, *dT *data set, dashed lines, *RH *data set). The data density is shown by the histogram in the top panel. The purple area marks the range of the shot noise for the range of size factors in the data set. One can see that the noise between technical replicates follows closely the shot noise limit, while the noise between biological replicates exceeds shot noise already for low count values.

This is confirmed by comparison of technical with biological replicates [1]. In Figure 7 we used *DESeq *to obtain variance estimates for the data of Nagalakshmi *et al. *[1]. The analysis indicates that the difference between technical replicates barely exceeds shot noise level, while biological replicates differ much more. Tests for differential expression that are based on a Poisson model, such as those discussed in References [6,7,20,22,23] should thus be interpreted with caution, as they may severely underestimate the effect of biological variability, in particular for highly expressed genes.

Consequently, it is preferable to use a model that allows for overdispersion. While for the Poisson distribution, variance and mean are equal, the negative binomial distribution is a generalization that allow for the variance to be larger. The most advanced of the published methods using this distribution is likely *edgeR *[8]. *DESeq *owes its basic idea to *edgeR*, yet differs in several aspects.

### Sharing of information between genes

First, we discovered that the use of total read counts as estimates of sequencing depth, and hence for the adjustment of observed counts between samples (as recommended by Robinson *et al. *[8] and others) may result in high apparent differences between replicates, and hence in poor power to detect true differences.

*DESeq *uses the more robust size estimate Equation (5); in fact, *edgeR*'s power increases when it is supplied with those size estimates instead. (Note: While this paper was under review, *edgeR *was amended to use the method of Oshlack and Robinson [13].)

For small numbers of replicates as often encountered in practice, it is not possible to obtain simultaneously reliable estimates of the variance and mean parameters of the NB distribution. *EdgeR *addresses this problem by estimating a single *common dispersion *parameter. In our method, we make use of the possibility to estimate a more flexible, mean-dependent local regression. The amount of data available in typical experiments is large enough to allow for sufficiently precise local estimation of the dispersion. Over the large dynamic range that is typical for RNA-Seq, the raw SCV often appears to change noticeably, and taking this into account allows *DESeq *to avoid bias towards certain areas of the dynamic range in its differential-expression calls (see Figure 2 and 4).

This flexibility is the most substantial difference between *DESeq *and *edgeR*, as simulations show that *edgeR *and *DESeq *perform comparably if provided with artificial data with constant SCV (Supplementary Note G in Additional file 1). *EdgeR *attempts to make up for the rigidity of the single-parameter noise model by allowing for an adjustment of the model-based variance estimate with the per-gene empirical variance. An empirical Bayes procedure, similar to the one originally developed for the *limma *package [24-26], determines how to combine these two sources of information optimally. However, for typical low replicate numbers, this so-called tagwise dispersion mode seems to have little effect (Figure 4) or even reduces *edgeR*'s power (Supplementary Note F in Additional file 1).

Third, we have suggested a simple and robust way of estimating the raw variance from the data. Robinson and Smyth [11] employed a technique they called quantile-adjusted conditional maximum likelihood to find an unbiased estimate for the raw SCV. The *quantile adjustment *refers to a rank-based procedure that modifies the data such that the data seem to stem from samples of equal library size. In *DESeq*, differing library sizes are simply addressed by linear scaling (Equations (2) and (3)), suggesting that quantile adjustment is an unnecessary complication. The price we pay for this is that we need to make the approximation that the sum of NB variables in Equation (10) is itself NB distributed. While it seems that neither the quantile adjustment nor our approximation pose reason for concern in practice, *DESeq*'s approach is computationally faster and, perhaps, conceptually simpler.

Fourth, our approach provides useful diagnostics. Plots such as Supplementary Figure S3 in Additional file 2 are helpful to judge the reliability of the tests. In Figure 1b and 7, it is easy to see at which mean value biological variability dominates over shot noise; this information is valuable to decide whether the sequencing depth or the number of biological replicates is the limiting factor for detection power, and so helps in planning experiments. A heatmap as in Figure 5 is useful for data quality control.

## Materials and methods

### The R package DESeq

We implemented the method as a package for the statistical environment R [27] and distribute it within the Bioconductor project [28]. As input, it expects a table of count data. The data, as well as meta-data, such as sample and gene annotation, are managed with the S4 class *CountDataSet*, which is derived from *eSet*, Bioconductor's standard data type for table-like data. The package provides high-level functions to perform analyses such as shown in Section *Application *with only a few commands, allowing researchers with little knowledge of R to use it. This is demonstrated with examples in the documentation provided with the package (the package vignette). Furthermore, lower-level functions are supplied for advanced users who wish to deviate from the standard work flow. A typical calculation, such as the analyses shown in Section *Applications*, takes a few minutes of time on a personal computer.

All the analyses presented here have been performed with *DESeq*. Readers wishing to examine them in detail will find a Sweave document with the commented R code of the analysis code as Additional file 2 and the raw data in Additional file 3.

*DESeq *is available as a Bioconductor package from the Bioconductor repository [28] and from [36].

## Abbreviations

ChIP-Seq: (high-throughput) sequencing of immunoprecipitated chromatin; ECDF: empirical cumulative distribution function; FDR: false-discovery rate; GLM: generalized linear model; RNA-Seq: (high-throughput) sequencing of RNA; SCV: squared coefficient of variation; NB: negative-binomial (distribution); VST: variance-stabilizing transformation.

## Authors' contributions

SA and WH developed the method and wrote the manuscript. SA implemented the method and performed the analyses.

## Supplementary Material

Additional file 1 **Supplement**. Contains all Supplementary Notes and Supplementary Figures. Click here for file

Additional file 2 **Supplement II**. PDF file presenting the source code of all the analyses presented in this paper, with comments, as a Sweave document. Click here for file

Additional file 3 **Raw data**. Tarball containing the raw data for the presented analyses. Click here for file
