# Supplementary material for: Differential expression analysis for sequence count data
Source: Genome Biol. 2010 Oct 27;11(10):R106. doi: 10.1186/gb-2010-11-10-r106 (PMC3218662; doi:10.1186/gb-2010-11-10-r106)
Supplement: Additional file 1 — Supplement. Contains all Supplementary Notes and Supplementary Figures. [file gb-2010-11-10-r106-S1.PDF]

Differential expression analysis for sequence count data

Simon Anders, Wolfgang Huber

Supplement

A Parametrisation of the negative binomial distribution

An integer valued random variable K is said to follow a negative binomial distribution with parameters $p \in]0,1[$ and $r \in]0,\infty[$ if [12]

$$\Pr(K=k) = \binom{k+r-1}{r-1} p^r (1-p)^k.$$

This two-parametric distribution can, equivalently, be parametrised in terms of its mean μ and variance σ^2, via

$$p = \frac{\mu}{\sigma^2} \quad \text{and} \quad r = \frac{\mu^2}{\sigma^2 - \mu}.$$

B Variance estimator

In Section , we claim that $\hat{w}_{i\rho} - z_{i\rho}$, as defined in Eqs. (7, 8), is an unbiased estimator for the raw variance $v_{i\rho}$. To show this, we compute the expectation value of $\hat{w}_{i\rho}$. To simplify notation, we suppress the indices i and ρ in the following. Furthermore, we neglect differences between the true library sizes s_j and their estimates $\hat{s}_j$. Then,

$$\hat{q} = \frac{1}{m} \sum_{j=1}^{m} \frac{K_j}{s_j}$$

is an unbiased estimator of q, because, due to Equation (2), $\mathbb{E}\, K_j = s_j q_0$. Next, we examine

$$(m-1)\, \hat{w} = \sum_{j:\, \rho_j = \rho} \left(\frac{k_j}{s_j} - \hat{q} \right)^2.$$

Taking expectations on both sides yields

$$(m-1)\, \mathbb{E}\, \hat{w} = \left(1 - \frac{1}{m}\right) \sum_j \frac{\mathbb{E}\, K_j^2}{s_j^2} - \frac{1}{m} \sum_{\substack{j,l \\ j \neq l}} \frac{\mathbb{E}\, K_j K_l}{s_j s_l}$$

For $j \neq k$, K_j and K_l are independent, and hence $\mathbb{E}\, K_j K_l = s_j s_l q^2$, while for $j = l$, we have $\mathbb{E}\, K_j^2 = (\mathbb{E}\, K_j)^2 + \operatorname{Var} K_j = s_j^2 q^2 + s_j q + s_j^2 v$ by the definition of variance and Equation (3). Using this, we find

$$\mathbb{E}\, \hat{w} = v + \underbrace{\frac{q}{m} \sum_j \frac{1}{s_j}}_{z},$$

where the under-braced part (with $\hat{q}$ plugged in for q) is the bias correction term z.

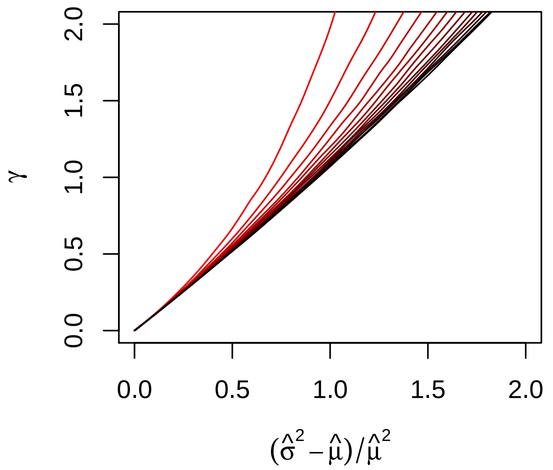

Figure S1: The bias adjustment function f_{mq}, for (red to black) $m = 2, 3, \ldots, 15$ in the limit of large q.

C Estimation biases

Plugging in the unbiased estimators for mean and variance of Section B into the expression for the density given in Section A leads to biased probabilities. As suggested by earlier work [29–32], and confirmed by our own simulations, approximately unbiased probabilities are obtained when, instead, unbiased estimates for mean and raw SCV are used.

This reparametrization from mean and variance to mean and raw SCV gives rise to new bias. We tabulated this bias for a range of parameters and use this for correction. The tabulation was done as follows.

Let f_{mq} be a function that maps a true raw SCV value γ to the expectation of the estimate $\hat{\gamma} = (\hat{\sigma}^2 - \hat{\mu})/\hat{\mu}^2$. $f_{mq}(\gamma)$ approaches its limit for $q \to \infty$ very fast; the changes for $q \gtrsim 30$ are negligible for our purposes, and the values for small q only lead to a conservative overestimation of the variance. Hence, we precalculate f_{mq} for a fixed, large value of q, and all the values $m = 2, 3, \ldots, 15$, at a grid of values for γ, invert it and interpolate in order to bias-correct an estimate $\hat{\gamma}$. (See Fig. S1 for a plot of the function f_{mq}.) For $m \gtrsim 15$, f_{mq} is sufficiently close to the identity function to make a bias correction unnecessary for our purposes.

D Numerical calculation of the p values

Evaluating the sums in Equation (11) requires some care. In HTS data, the count sum k_S can be large (e.g., millions

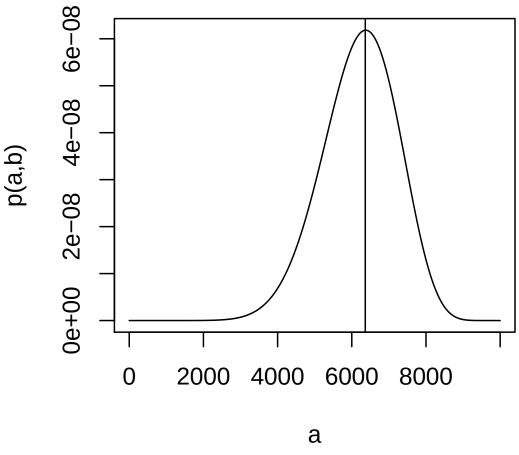

Figure S2: Shape of the function $p(a,b)$, with $k_{\mathrm{S}} = 10{,}000$, $b = k_{\mathrm{AB}} - a$, $\mu_{\mathrm{A}} = 7{,}000$, $\mu_{\mathrm{B}} = 4{,}000$, $\sigma^2_{\mathrm{A}} = \mu_{\mathrm{A}} + 0.1\mu^2_{\mathrm{A}}$ and $\sigma^2_{\mathrm{B}} = \mu_{\mathrm{B}} + 0.1\mu^2_{\mathrm{B}}$. The vertical line marks the estimate $k_{\mathrm{S}}\mu_{\mathrm{A}}/(\mu_{\mathrm{A}} + \mu_{\mathrm{B}})$ for the mode.

of counts for a single strongly expressed gene), and calculating all the summands individually may take a long time and result in rounding error accumulation. Figure S2 shows the dependence of $p(a,b)$ (as defined in Section and using Equation (14) for the distribution of K_{A}) on a for typical parameters. The function is unimodal, with mode approximately at ratio a/b equal to the ratio of the means of K_{A} and K_{B}. The function's simple shape allows the following numerical approximation: start at evaluating the sum from the peak (or rather, from its estimated location according to the means) and proceed outwards in two passes, first left, then right. During the summation, watch the changes of the value and keep adapting the step size according to a pre-defined precision goal. The value of p for the observed count values k_{A} and k_{B} is calculated beforehand, so that both the sum in the numerator and denominator of Equation (11) can be calculated in the same pass. To compute the density of the NB distribution, we use a function [33] in the C API of R [27].

E Diagnostics for the local regression

The choice of the gamma family for the local regression can be motivated as follows: If the size-adjusted counts k_{ij}/s_j in the sample variance estimate $w_{i\rho}$ calculated in Equation (7) were normally distributed with true variance σ^2_{ij}, the quantity $(m_\rho - 1)w_{i\rho}/\sigma^2_{ij}$ would follow a χ^2 distribution with $m_\rho - 1$ degrees of freedom, and this should hold as well for the residuals,

$$\xi_{i\rho} = (m_\rho - 1)\frac{w_{i\rho}}{w(\hat{q}_{i\rho})}$$

(where we have replaced the true variance σ^2 with its fitted value $w(\hat{q}_{i\rho})$. Even though the size-adjusted counts

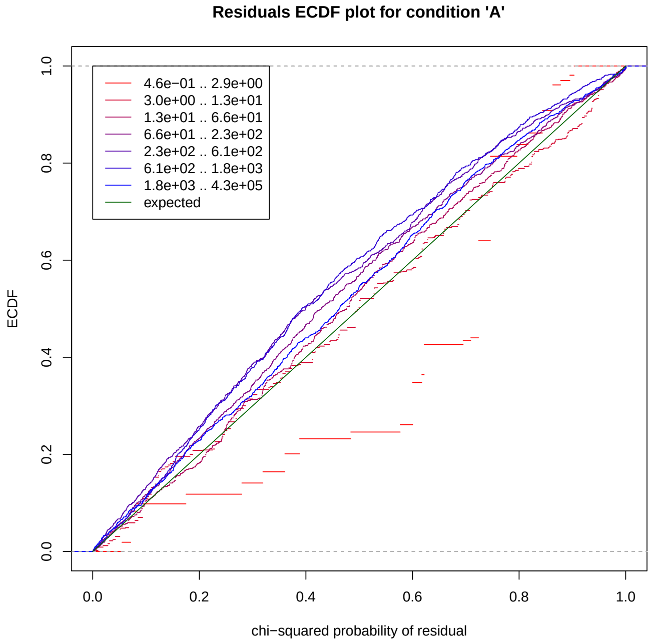

Figure S3: Empirical cumulative density function (ECDF) plots for the χ^2-probabilities of the residuals from the variance fit (orange line in Figure 1), stratified by the mean. The green line is the diagonal, which is the expected curve if the residuals follow the χ^2 distribution with $m_\rho - 1 = 1$ degree of freedom.

are not normally distributed, this is still a useful approximation for GLM local regression. Among the exponential families commonly used with generalised linear models, the gamma family, which includes the χ^2 distributions, is close to the actual distribution of the residuals, and since generalised linear models tend to show robustness against misspecification, we expect a reasonable fit. In order to verify this, we can check how well the residuals $\xi_{i\rho}$ follow a χ^2 distribution. To this end, we calculate the χ^2 quantiles of the $\xi_{i\rho}$ and check them for uniformity by plotting their empirical cumulative density function (ECDF). Figure S3 shows the ECDF curves for the condition ρ = GNS, stratified by the estimated means $\hat{q}_{i\rho}$. As one can see, the residuals follow the distribution reasonably well. Only for extremely low counts (below 5), the fitting quality is reduced. At such low counts, the shot noise dominates (see Figure 1b), and inaccuracies in the estimation of the raw noise are not a reason for concern.

It is worth noting that the χ^2 distribution for $m_{\mathrm{GNS}} - 1 = 1$ degree of freedom has a heavy right tail. Hence, the fact that in Figure 1 so many points lie far above the fitted line does not imply a bad fit.

F Differential expression analysis for the neural stem cell data set

In the main text, we have demonstrated our method with an RNA-Seq data set from *Drosophila* embryos. To show

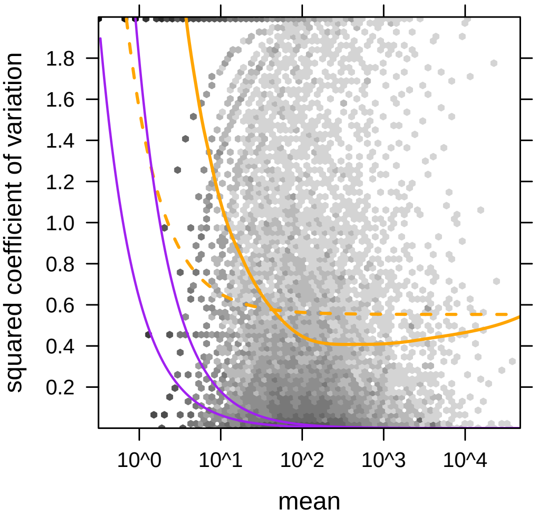

Figure S4: Estimation of the SCV for the neural cell data. Compare with the fly data (Figures 1 and S9. See caption to Figure 1 for further description.)

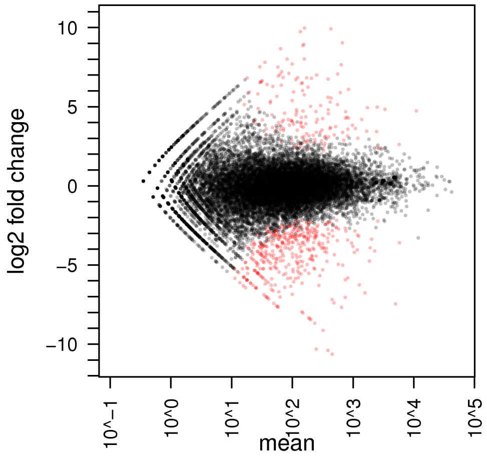

Figure S5: Testing for differential expression between conditions *GNS* and *NS* in the neural cell data. Compare to Figure 3.

that *DESeq* is flexible in dealing with very different noise types, we performed the same analysis here for the Tag-Seq data set by Engström et al. [18]. The data set comprises of four tissue cultures derived from glioblastoma-derived neural stem cells (condition “GNS”) with two tissue cultures derived from non-cancerous neural stem cells (condition “NS”).

The number of reads obtained from each library varied from 7.6 millions to 13.6 millions. A good fraction of these (depending on the sample, from 32% to 53%) could be unambiguously assigned to annotated genes, and Engström et al. summarised the data in a table of counts with six columns for the six samples and 18,760 rows, one for each gene. For the differential expression analysis, we use only two of the four GNS samples (excluding one with a different histopathology and using only one of two samples taken from the same patient).

Each of these samples is taken from a different sub-

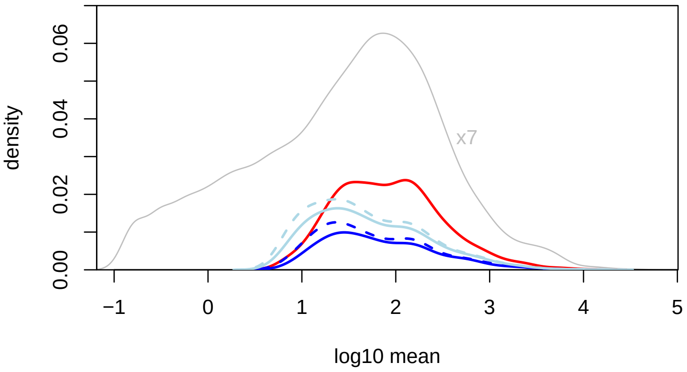

Figure S6: Same plot as Figure 4, now for the neural cell data. Again, red is *DESeq*’s and blue *edgeR*’s result. (light blue, using read count sum for library size adjustment; dark blue, using Equation (5); solid, with common dispersion; dashed, with tagwise dispersion.)

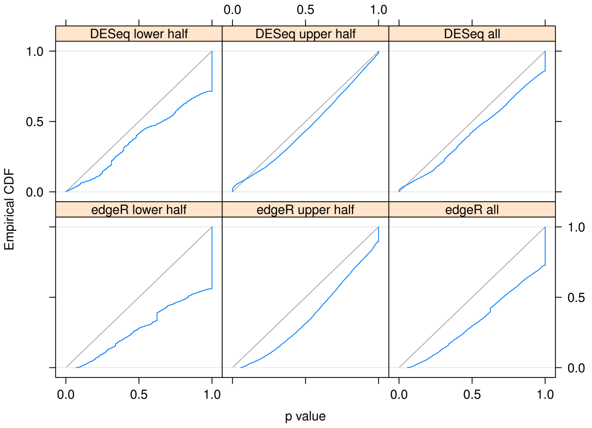

Figure S7: Type-I error control for the neural cell data. Compare with Figure 2.

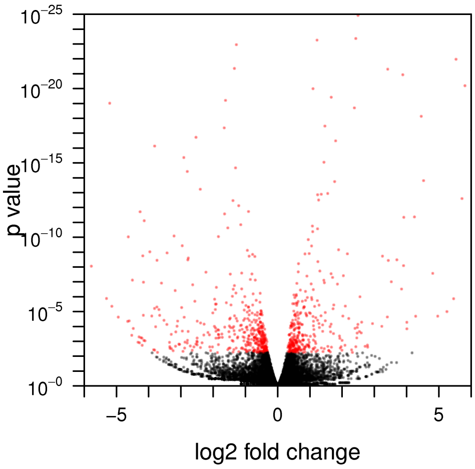

Figure S8: Volcano plot for the comparison of conditions *A* and *B* in the fly RNA-Seq data.

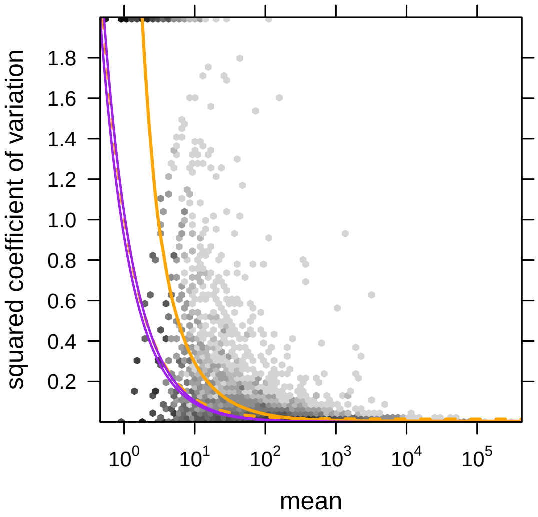

Figure S9: The SCV plot for the fly data, i.e., the same plot as in Fig. 1b, but here with the y axis spanning the whole data range.

ject and hence has a different genetic background, giving rise to strong variability. This explains why in the SCV plot for this data set is by orders of magnitude stronger than in the fly case. (Compare Figure S4 with Figure S9.) Nevertheless, a test for differential expression yields useful results: at 10% FDR, *DESeq* calls significant differences for 673 of the 18,392 genes with non-zero counts (Figure S5).

We also compared the two *GNS* samples with the two *NS* samples using *edgeR*. *EdgeR* finds 452 genes when used in *common dispersion* mode and 256 in the *tagwise dispersion* mode. These numbers for *edgeR* were obtained when supplying it with total read counts as library size parameters, as recommended in the documentation; when *DESeq*'s estimates, as in Equation (5), were used, we obtained 525 and 316 genes, respectively. 84% to 96% of *edgeR*'s genes were also reported by *DESeq*, which is consistent with an FDR of 10%.

As with the fly data, the difference between the results of *edgeR* and *DESeq* does not merely lie in the number of genes, but also in their distribution along the abundance scale. We can recover all the observation we made for the fly data: Figure S6 shows that, again, *edgeR*'s hits tend to concentrate at lower abundance, while the hits from *DESeq* are more evenly distributed along the dynamic range, once the mean is above ~ 10. This agrees with Figure S4: *edgeR*'s estimate for the common dispersion (0.56), as indicated by the dashed orange line in Figure S4 is lower than *DESeq*'s estimate (solid orange line) for the lower part of the the dynamic range, and higher in the upper range, causing *edgeR* to calls more hits among genes with low counts and be too conservative for genes with high counts. Also, a comparison of the two GNS replicates against each other, shows the same effect: As can be seen in Figure S7, there are either too many high or too many low p values, depending on the range of mean values.

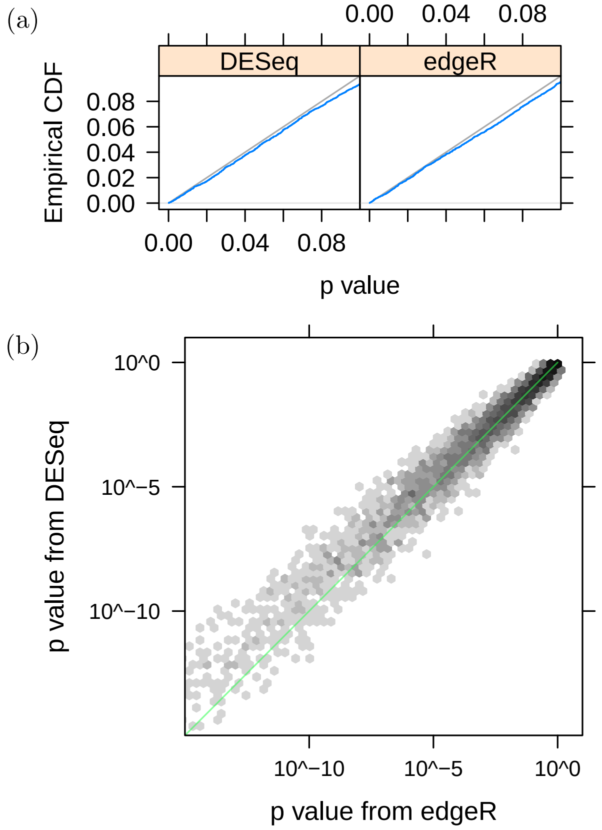

Figure S10: Simulation results (for constant raw SCV). (*a*) Uniformity of the p values calculated for the genes that were not differentially expressed, shown with an ECDF plot. (*b*) Comparison of the p values between the *DESeq* and *edgeR* for the genes that were simulated as differentially expressed.

G Simulations

This section describes simulations to assess how *DESeq* and *edgeR* perform when the data are generated exactly by the assumed model. We ran multiple simulations with different parameters. Here we discuss results from two simulation runs with typical settings. In the first run, we followed *edgeR*'s model, which assumes a constant raw SCV, while in second run, we let the raw SCV depend on the mean, in order to test *DESeq*'s more general noise model.

We drew true mean values q_i for 20,000 genes from an exponential distribution with rate 1/250. Each gene was considered "truly differentially expressed" (tDE) with probability 30%, and for all tDE genes a $\log_2$ fold change was randomly drawn from a normal distribution with mean 0 and standard deviation 0.7. Finally, four count values were drawn for each gene, two for condition A and two for condition B, from negative binomial distributions, with the given means, and with variances as described below, and multiplied by the size factors, which we chose as 0.5, 1.7, 1.4 and 0.9.

For the variances, we first catered to *edgeR*'s assumption and set the raw SCV to a constant, 0.015. (All these values were chosen to mimic the situations in the

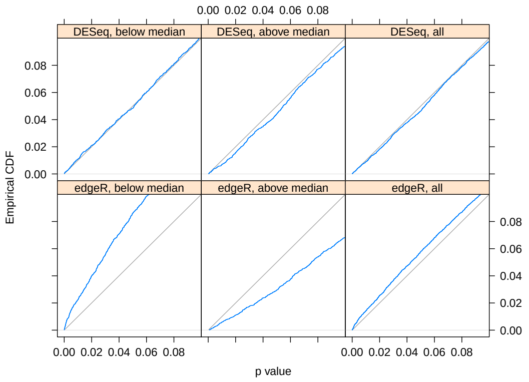

Figure S11: Simulation results (for mean-dependent raw SCV): ECDF curves for the p values from not differentially expressed genes for *DESeq* (upper row) and *edgeR* (lower row), stratified by mean (lower and upper half in left and middle column), and using full data (right column).

real data sets described in the main text.)

We used both *DESeq* and *edgeR* to test for differential expression. *edgeR* was given the true size factors and was allowed to assume that the dispersion is common between the conditions. *DESeq* had to estimate the size factors from the data. Both *edgeR* and *DESeq* estimated the raw SCV correctly and with good accuracy. Both approaches controlled the type-I error rate correctly: the p values of the non-tDE genes follow a uniform distribution (Fig. S10a). Also, the p value distributions of tDE genes, and hence the detection power, were very similar between the two methods, as can be seen in Fig. S10b.

This simulation assumed *edgeR*'s model of constant dispersion. *DESeq*'s main advantage over *edgeR* is the flexible variance estimation that is not needed here. This is why both methods performed equally well.

If we let the raw SCV depend on the common-scale mean, *DESeq* and *edgeR* perform differently. For Fig. S11, a simulation was performed with the same parameters as above, except for the raw SCV, which we varied by one decade, from 0.1 to 0.01, over the range of common-scale mean values by assigning to it the sigmoid function $\alpha(\mu) = .01 + 9/(\mu + 100)$.

Fig. S11 shows the p values for the not differentially expressed genes (true negatives). In the same manner as observed for the examined real data sets, *edgeR* maintains proper type-I error control, but only when considering all genes together. If one stratifies by mean, i.e., looks at weakly and strongly expressed genes separately, *edgeR* fails to maintain type-I error control. This was despite the fact that we enabled *edgeR*'s tagwise dispersion mode for this simulation, which is meant to deal with non-constant dispersion, but seemingly was not effective for the small number of replicates that we simulated. *DESeq*, designed for data with intensity-dependent noise, maintains type-I error control for each value range independently.
