# Supplementary material for: Differential expression analysis for sequence count data
Source: Genome Biol. 2010 Oct 27;11(10):R106. doi: 10.1186/gb-2010-11-10-r106 (PMC3218662; doi:10.1186/gb-2010-11-10-r106)
Supplement: Additional file 2 — Supplement II. PDF file presenting the source code of all the analyses presented in this paper, with comments, as a Sweave document. [file gb-2010-11-10-r106-S2.PDF]

Differential expression analysis for sequence count data

Simon Anders, Wolfgang Huber

Supplement II

In order to make the analyses presented in our paper transparent and reproducible, we document in the present supplement the complete R code used to perform them and to create all the plots in the paper and in Supplement I. The purpose of this document is *not* to explain the use of the *DESeq* package; for this, please read the vignette (manual) supplied with the package.

1 Required packages

We start with loading all requried packages:

```
> library( DESeq )
> library( edgeR )
> library( hexbin )
> library( latticeExtra )
> library( gplots )
> library( geneplotter )
```

This Sweave file has been run with these package versions:

```
> sessionInfo()

R version 2.12.0 Under development (unstable) (2010-08-22 r52792)
Platform: x86_64-unknown-linux-gnu (64-bit)

locale:
 [1] LC_CTYPE=en_US.UTF-8       LC_NUMERIC=C
 [3] LC_TIME=en_US.UTF-8        LC_COLLATE=en_US.UTF-8
 [5] LC_MONETARY=C              LC_MESSAGES=en_US.UTF-8
 [7] LC_PAPER=en_US.UTF-8       LC_NAME=C
 [9] LC_ADDRESS=C               LC_TELEPHONE=C
[11] LC_MEASUREMENT=en_US.UTF-8 LC_IDENTIFICATION=C

attached base packages:
[1] grid      stats     graphics  grDevices utils     datasets  methods
[8] base

other attached packages:
 [1] geneplotter_1.27.0   annotate_1.27.1      AnnotationDbi_1.11.4
 [4] gplots_2.8.0         caTools_1.10         bitops_1.0-4.1
 [7] gdata_2.8.0          gtools_2.6.2         latticeExtra_0.6-14
[10] RColorBrewer_1.0-2   hexbin_1.22.0        edgeR_1.5.11
[13] DESeq_1.1.12         locfit_1.5-6         lattice_0.18-8
[16] akima_0.5-4          Biobase_2.9.0
```

```
loaded via a namespace (and not attached):
[1] DBI_0.2-5        genefilter_1.31.2 limma_3.5.18      RSQLite_0.9-2
[5] splines_2.12.0    survival_2.35-8   xtable_1.5-6
```

2 Reading in the data

All data files necessary to run the code shown here are supplied in the supplementary data tarball available with the paper.

2.1 Fly RNA-Seq data

The RNA-Seq data from gene overexpression in fly embryos is unpublished, an excerpt of which was kindly provided to us by Wilczynski et al. (Furlong Lab, EMBL Heidelberg) in the form of the following table (file `fly_RNA_counts.tsv`, supplied with supplementary data):

```
> countsTableFly <- read.delim( "fly_RNA_counts.tsv" )
> condsFly <- c( "A", "A", "B", "B" )
> # Add dummy names to avoid confusion later
> rownames( countsTableFly ) <- paste( "Gene", 1:nrow(countsTableFly), sep="_" )
```

2.2 Neural stem cell RNA-Seq data

The RNA-Seq data comparing neural stem cell lines from gliablastomas and non-cancerous tissue was kindly provided to us by Engström et al. (Bertone Lab, EMBL-EBI Hinxton) in the form of the following table (file `NeuralStemCellData.tab`, supplied with supplementary data):

```
> countsTableNS <- read.delim( "NeuralStemCellData.tab", row.names=1 )
> head(countsTableNS)
```

```
         GliNS1 G144 G166 G179 CB541 CB660
13CDNA73      4    0    6    1     0     5
15E1.2       75   74  222  458   215   167
182-FIP     118  127  555  231   334   114
2'-PDE       39   38   98  127    34    40
3'HEXO       18   20   76  111   121   112
3.8-1         0    0    1    0     0     0
```

The conditions are

```
> condsNS <- c( "GNS_dupe", "GNS", "GNS", "GNSL", "NS", "NS" )
```

where the first entry is for the sample that will be eliminated later as it is derived from the same patient as the second one.

2.3 Yeast RNA-Seq data

We have downloaded the deposited reads for the yeast RNA-Seq experiment by Nagalakshmi et al. from the NCBI Short Read Archive, remapped them to the yeast genome with Bowtie (Ref. [34] in the paper) and the counted the coverage of each gene with htseq-count (part of HTSeq, Ref. [35] in the paper).

We read in this data with

```
> countsYeast <- read.delim( "counts_Nagalakshmi_et_al.tab", row.names=1 )
> head( countsYeast )

         dT_bio dT_ori dT_tech RH_bio RH_ori RH_tech
15S_rRNA      6     20      31     79     51      65
21S_rRNA    531    530     755    932    285     369
HRA1          1      0       0      1      2       3
LSR1          3      3      12    371    126     202
NME1          3      5       7      9      7       7
Q0010         0      1       4      0      0       0
```

3 ChIP-Seq data

The supplementary file `PolII_counts.tsv` contains one column for each sample in the Kasowski et al. paper, and one row for each polymerase-II binding region that the authors identified, giving the number of reads counted to map within each binding region. This file has been derived from the original data file in Kasowski et al.'s supplementary data with a simple reformatting.

We read in the data with

```
> countsPolII <- read.delim( "PolII_counts.tsv" )
> # make rownames
> row.names(countsPolII) <- sprintf( "%s:5d-%d", countsPolII$chrom,
+    countsPolII$start, countsPolII$stop )
> # The IDs should start with "GM", not "X":
> colnames(countsPolII) <- sub( "X", "GM", colnames(countsPolII) )
> # A few individuals in this data file have four or more replicates. We have picked
> # (at random) two of them:
> countsPolII <- countsPolII[ , grepl( "GM12878", colnames(countsPolII) ) |
+    grepl( "GM12891", colnames(countsPolII) ) ]
> head(countsPolII)

               GM12878_1 GM12878_2 GM12878_3 GM12878_4 GM12878_6 GM12878_7
chr1:5d-703615       108        71        74        82       119       139
chr1:5d-752153        32        31        25        29        31        21
chr1:5d-829805        46        41        26        38        12        21
chr1:5d-884146        44        41        27        54        29        30
chr1:5d-885683        17         9         7         8         5         6
chr1:5d-891561        32        16        13        21        10        20
               GM12891_1 GM12891_2 GM12891_3 GM12891_4 GM12891_5 GM12891_6
chr1:5d-703615        68       126       116        56        51        49
chr1:5d-752153        16        16        14        10        14        14
chr1:5d-829805        23        34        25        11        11        12
chr1:5d-884146        35        68        37        11        20        27
chr1:5d-885683         7        12        12         2         2         4
chr1:5d-891561        17        27        24        18         9        10
```

4 Analysis of fly data

4.1 Analysis with DESeq

For the standard DESeq analysis, we instantiate a *CountDataSet*, then estimate the size factors and the variance functions, and finally perform the binomial test.

```
> cdsFly <- newCountDataSet( countsTableFly, condsFly )
> cdsFly <- estimateSizeFactors( cdsFly )
> cdsFly <- estimateVarianceFunctions( cdsFly )
> resFly <- nbinomTest( cdsFly, "A", "B" )
```

4.2 Analysis with edgeR

For the edgeR analysis, we test for different modes: One choice is whether edgeR should run in the common ("C") or the tag-wise ("T") dispersion mode, the other is whether edgeR should be given the actual number of sequenced reads ("N"), as suggested in the edgeR manual, or the same size factors[1] as DESeq used ("D").

We instantiate four *DGEList* objects and perform the analysis four times:

```
> # using DESeq's size factors:
>
> dglFlyD <- DGEList( counts=countsTableFly, group=condsFly,
+    lib.size=1e7*sizeFactors(cdsFly) )
> dglFlyD <- estimateCommonDisp( dglFlyD )
> dglFlyD <- estimateTagwiseDisp( dglFlyD )

Using grid search to estimate tagwise dispersion.

> # test with tag-wise dispersion:
>
> edgerResFlyDT <- exactTest( dglFlyD, common.disp=FALSE )

Comparison of groups:  B - A

> edgerResFlyDTPadj <- p.adjust( edgerResFlyDT$table$p.value, method="BH" )
> # common dispersion, DESeq's size factors:
>
> edgerResFlyDC <- exactTest( dglFlyD, common.disp=TRUE )

Comparison of groups:  B - A

> edgerResFlyDCPadj <- p.adjust( edgerResFlyDC$table$p.value, method="BH" )
> # with the original library sizes:
>
> # the total number of sequenced counts were as follows:
> libsizesFly <- c( A1=13613446, A2=12106517, B1=12920058, B2=11599459 )
> dglFlyN <- DGEList( counts=countsTableFly, group=condsFly,
```

[1]As edgeR expects read counts, we multiply these by 10^7 so that they get a plausible magnitude. edgeR only cares about the relative ratios of the `lib.size` values, and hence, this factor is unimportant. However, it expect them to be large numbers (it seems to round them to integers), and hence, the "`1e7*`" below ensures that no information is lost in rounding. Using another large number does not change the results.

```
+    lib.size=libsizesFly )
> dglFlyN <- estimateCommonDisp( dglFlyN )
> dglFlyN <- estimateTagwiseDisp( dglFlyN )
```

```
Using grid search to estimate tagwise dispersion.
```

```
> # test with tag-wise dispersion:
>
> edgerResFlyNT <- exactTest( dglFlyN, common.disp=FALSE )
```

```
Comparison of groups:  B - A
```

```
> edgerResFlyNTPadj <- p.adjust( edgerResFlyNT$table$p.value, method="BH" )
> # common dispersion, DESeq's size factors:
>
> edgerResFlyNC <- exactTest( dglFlyN, common.disp=TRUE )
```

```
Comparison of groups:  B - A
```

```
> edgerResFlyNCPadj <- p.adjust( edgerResFlyNC$table$p.value, method="BH" )
```

4.3 Plots and comparison

4.3.1 Figures on variance estimation

We start with the plots of Fig. 1 in the paper.

The common dispersion values estimated by edgeR are:

```
> dglFlyD$common.dispersion
```

```
[1] 0.01420442
```

```
> dglFlyN$common.dispersion
```

```
[1] 0.01436905
```

The values for the two library size settings hardly differ, we shall use the first one for the dashed orange line.

```
> alpha <- dglFlyD$common.dispersion
```

The *DESeq* package offers the function `rawVarFunc`, that returns the fitted raw variance function for a condition ρ, i.e., the function $\hat{v}_\rho(q)$ defined in Eq. (9) of the paper. For the solid orange line in the plot, we define a function `basevarFunc` that returns the fitted variance function $w_\rho(q)$ by undoing the subtraction of Eq. (9) that `rawVarFunc` does internally:

```
> baseVarFunc <- function( cds, cond ) {
+    rvf <- rawVarFunc( cds, cond )
+    sf <- sizeFactors(cds)[ conditions(cds) == cond ]
+    xim <- sum(1/sf) / length(sf)
+    function( q ) rvf( q ) + xim * q
+ }
```

We also need the gene-wise variance estimates (for the scatter plot part of Fig. 1)

```
> diagForA <- varianceFitDiagnostics( cdsFly, "A" )
```

The scatter points in the plots are actual hexagonal bins, produced with the *hexbin* package. While this looks nice, it unfortunately requires a rather involved call to routines from the *lattice* to get a proper log-log plot:

```
> # pdf( "var_mean_fly.pdf", width=4, height=4 )
>
> # two functions to make nice exponents for the axis labels
>
> xscale.components.log <- function( ... ) {
+    res = xscale.components.default( ... )
+    res$bottom$labels$labels = do.call( expression,
+       lapply( res$bottom$labels$at, function(a)
+          substitute( 10^b, list(b=a) ) ) )
+    res
+ }
> yscale.components.log = function( ... ) {
+    res = yscale.components.default( ... )
+    res$left$labels$labels = do.call( expression,
+       lapply( res$left$labels$at, function(a)
+          substitute( 10^b, list(b=a) ) ) )
+    res
+ }
> xg <- seq( log10(1/3),
+    log10(max( diagForA$baseMean )), length.out=1000 )
> print( xyplot(
+    baseVar ~ baseMean,
+    diagForA[ diagForA$baseMean>0,],
+    panel = function( ... ) {
+       panel.hexbinplot( ... )
+
+       # The solid orange line with the fitted base variance
+       # (Note that we have to manually switch between natural
+       # and logarithmic axis scaling, because hexbin cannot deal
+       # with automatic logarithmic scaling):
+
+       llines( xg, log10( baseVarFunc(cdsFly,"A")( 10^xg ) ),
+          col="orange", lwd=2 )
+
+       # The dashed orange line with edgeR's variance fit,
+       # i.e., variance = mean + alpha * mean^2:
+
+       llines( xg, log10( 10^xg + alpha * (10^xg)^2 ),
+          col="orange", lwd=2, lty="dashed" )
+
+       # For each sample with condition "A", plot a purple line
+       # with the shot noise, variance = mean, where the mean
```

```
+       # needs to be scaled by the size factor:
+
+       for( sf in sizeFactors( cdsFly )[ conditions(cdsFly) == "A" ] )
+          llines( xg, log10( 10^xg*sf ), col="purple", lwd=1.5 ) },
+
+    trans=function(x) x^(1/6),
+    inv=function(x) x^6,
+    xbins=80,
+    scales = list(
+       x = list( log=TRUE, axs="i" ),
+       y = list( log=TRUE, axs="i", limits=c( 3e-5,3e8 ), tick.number=8 ) ),
+    xlab="mean", ylab="variance",
+    xscale.components = xscale.components.log,
+    yscale.components = yscale.components.log
+ ) )
```

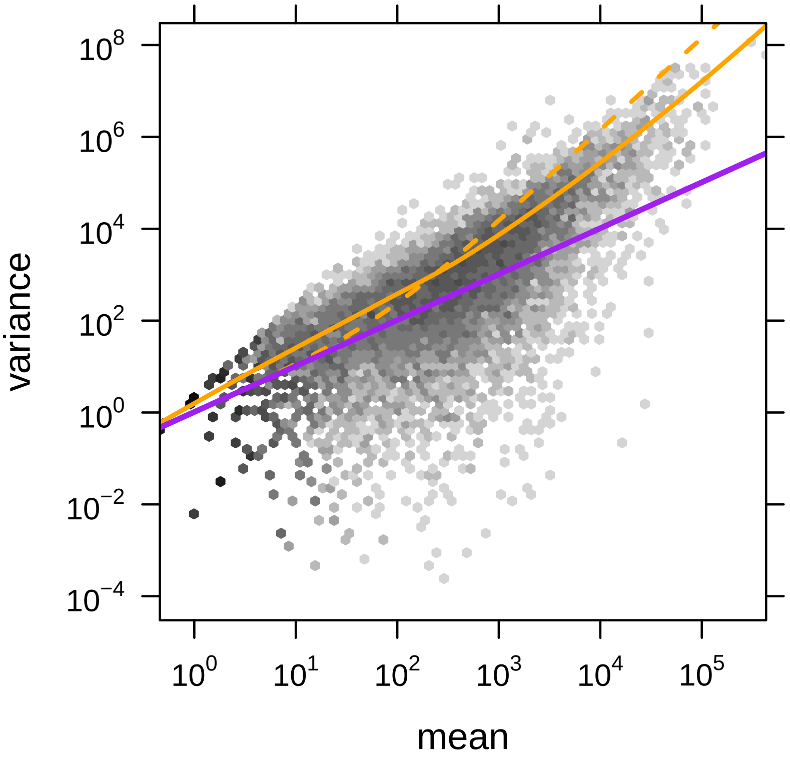

For Fig. 1b, the code is quite similar:

```
> # pdf( "cv2_mean_fly.pdf", width=4, height=4 )
>
> print(xyplot(
+    I( baseVar/baseMean^2 ) ~ baseMean,
```

```
+    diagForA[ diagForA$baseMean>0,],
+    panel = function( ... ) {
+       panel.hexbinplot( ... )
+
+       # As we convert from a variance to an SCV, we should adjust for
+       # bias, as described in Supplementary Note C. The function
+       # adjustScvForBias takes care of this. It requires, as second
+       # argument, the information how many samples were used for the
+       # variance estimate. The 'attr(...)' expression supplies this,
+       # returning in this case simply the value 2:
+
+       llines( xg,
+          adjustScvForBias(
+             baseVarFunc(cdsFly,"A")( 10^xg ) / (10^xg)^2,
+             attr( rawVarFunc(cdsFly,"A")(NA), "size" ) ),
+          col="orange", lwd=2 )
+
+       # The other lines are as before, only without the log of
+       # the y value but with a division by mean^2.
+
+       llines( xg, ( 10^xg + alpha * (10^xg)^2 ) / (10^xg)^2,
+          col="orange", lwd=2, lty="dashed" )
+
+       for( sf in sizeFactors( cdsFly )[ conditions(cdsFly) == "A" ] )
+          llines( xg, 1 / (10^xg * sf), col="purple", lwd=1.5 ) },
+
+    trans = function(x) x^(1/4),
+    inv = function(x) x^4,
+    xbins = 80,
+    scales = list(
+       x = list( log=TRUE, axs="i" ),
+       y = list( log=FALSE, axs="i", tick.number=8, limits=c(0,.2) ) ),
+    xlab = "mean", ylab="squared coefficient of variation",
+    xscale.components = xscale.components.log,
+ ))
```

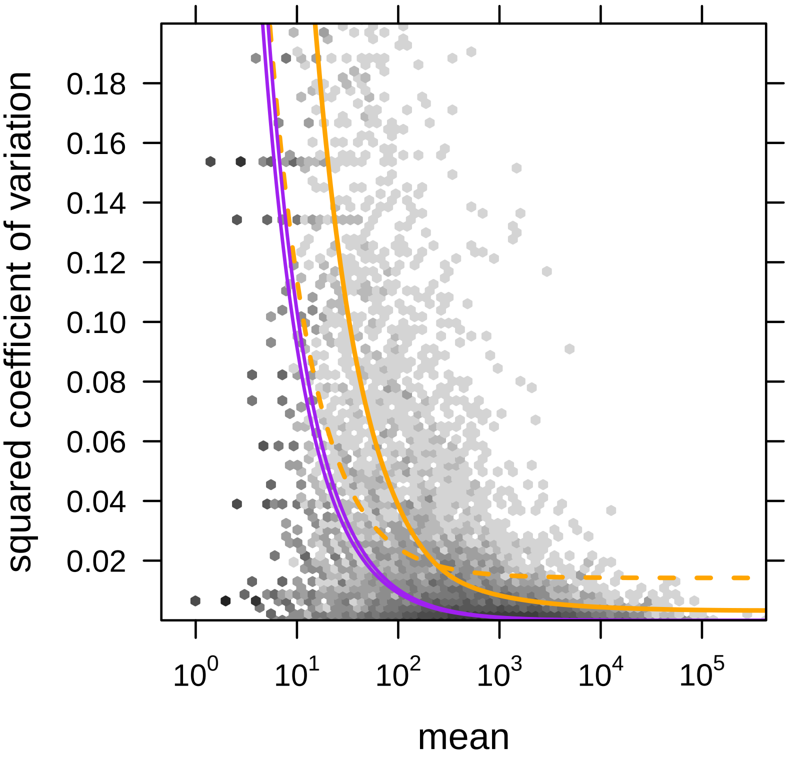

Just for completeness, the code for Suppl. Fig. S9, which is nearly identical to the previous code block:

```
> # pdf( "cv2_mean_full_fly.pdf", width=4, height=4 )
>
> print(xyplot(
+    I( baseVar/baseMean^2 ) ~ baseMean,
+    diagForA[ diagForA$baseMean>0,],
+    panel = function( ... ) {
+       panel.hexbinplot( ... )
+       llines( xg,
+          adjustScvForBias(
+             baseVarFunc(cdsFly,"A")( 10^xg ) / (10^xg)^2,
+             attr( rawVarFunc(cdsFly,"A")(NA), "size" ) ),
+          col="orange", lwd=2 )
+       llines( xg, ( 10^xg + alpha * (10^xg)^2 ) / (10^xg)^2,
+          col="orange", lwd=2, lty="dashed" )
+       for( sf in sizeFactors( cdsFly )[ conditions(cdsFly) == "A" ] )
+          llines( xg, 1 / (10^xg * sf), col="purple", lwd=1.5 ) },
+    trans = function(x) x^(1/4),
+    inv = function(x) x^4,
```

```
+    xbins = 80,
+    scales = list( x = list( log=TRUE, axs="i" ),
+       y = list( log=FALSE, axs="i", tick.number=8 ) ),
+    xlab = "mean", ylab="squared coefficient of variation",
+    xscale.components = xscale.components.log
+ ))
```

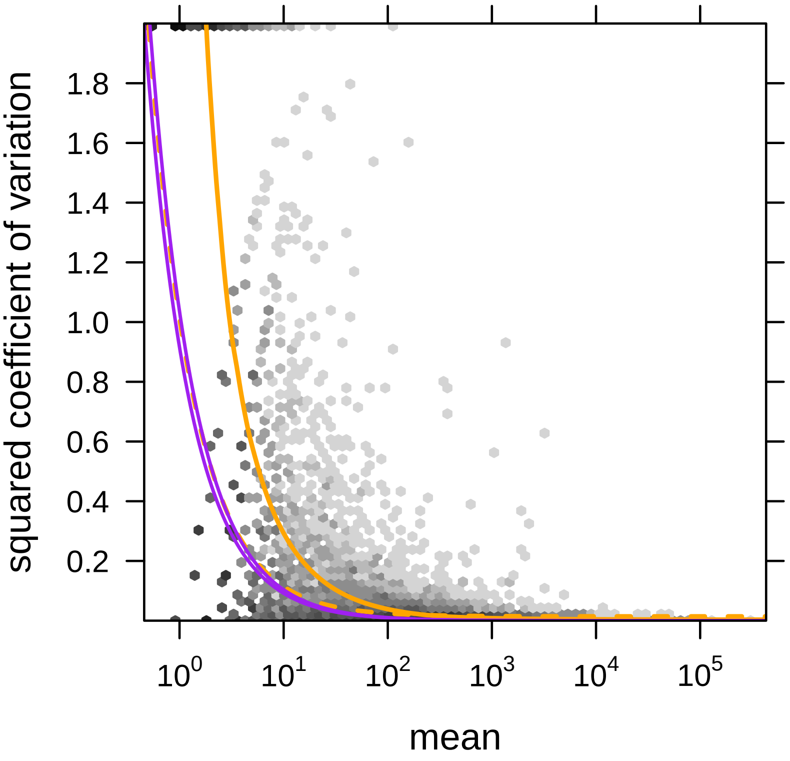

Finally, for the residuals ECDF plot (Fig. S3), the apropriate function of DESeq was used:

```
> # pdf( "resEcdf_A_fly.pdf", width=8, height=8 )
>
> residualsEcdfPlot( cdsFly, "A" )
```

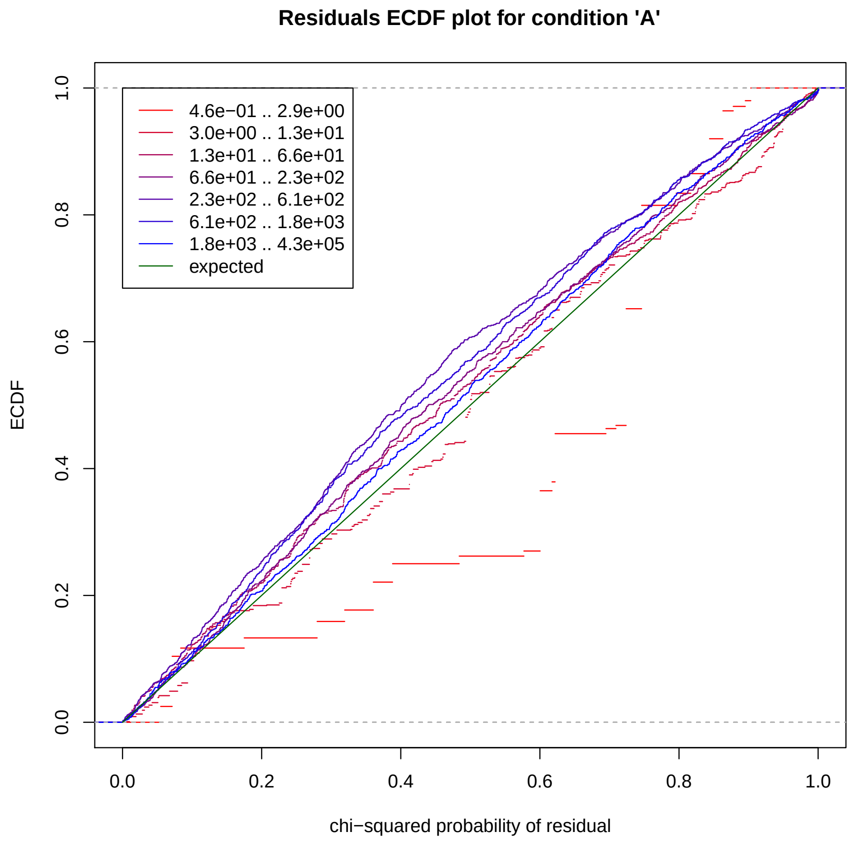

4.4 Figures for DE analysis

For Fig. 3, we plot one dot for each gene, using semi-transparent colour values (i.e., specified with alpha channel) in order to make overplotting of dots apparent. (The *hexbin* package cannot be used as we have two colours, black and red, to indicate significance.) Extra code, in the form of a panel function, is needed in order to draw the small arrows indicating genes with fold changes outside the displayed value range. We also customise the axis function to get nicer labels.

```
> # pdf( "DE_fly.pdf", width=4, height=4 )
>
> print(xyplot(
+    log2FoldChange ~ I( baseMean ),
+    resFly,
+    pch=16, cex=.3,
+    col=ifelse( resFly$padj < .1, "#FF000040", "#00000040" ),
+    panel = function( x, y, col, ... ) {
+       above <- (y > 5.8)
+       below <- (y < -5.8)
+       inside <- !( above | below )
+       panel.xyplot( x=x[inside], y=y[inside], col=col[inside], ... )
+       panel.arrows( x[above], 5.8, x[above], 5.95, col=col[above],
```

```
+          length=".1", unit="native" )
+       panel.arrows( x[below], -5.8, x[below], -5.95, col=col[below],
+          length=".1", unit="native" ) },
+    axis = function( side, ... ) {
+       if( side=="left") {
+          panel.axis( side, outside=TRUE, at=seq(-14,14,by=1), labels=FALSE )
+          panel.axis( side, outside=TRUE, at=seq(-10,10,by=5), labels=TRUE )
+       }
+       if( side=="bottom") {
+          panel.axis( side, outside=TRUE, at=seq(-2,10,by=1), rot=0,
+             labels = do.call( expression,
+                lapply( seq(-2,10,by=1), function(a)
+                   substitute( 10^b, list(b=a) ) ) ) ) )
+       } },
+    xlab = "mean", ylab = "log2 fold change",
+    scales = list(
+       x = list( log=TRUE ),
+       y = list( log=FALSE, limits=c( -6, 6 ) ) ) ))
```

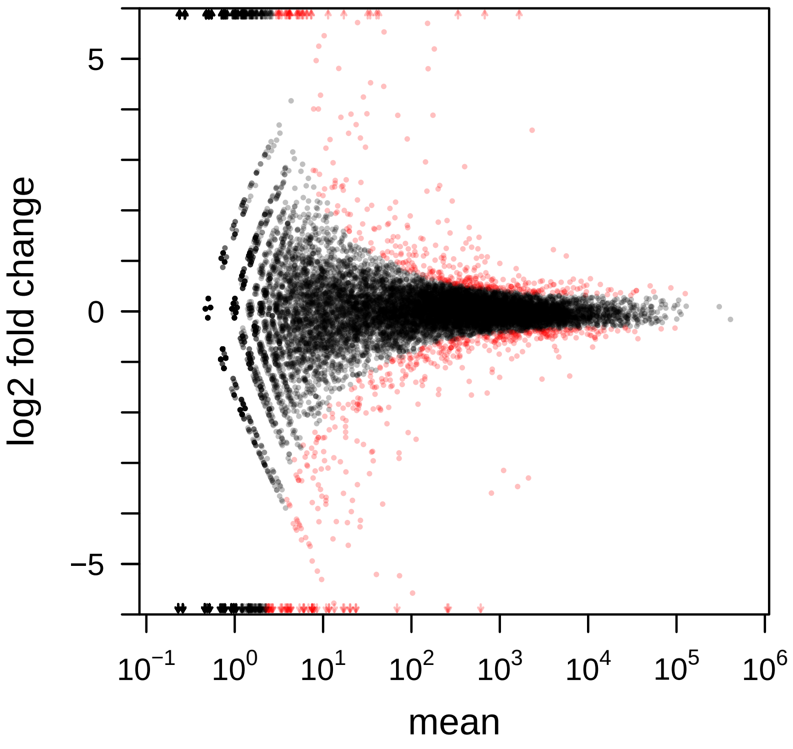

The volcano plot (Fig. S8) has been produced with:

```
> # pdf( "vulcano_fly.pdf", width=4, height=4 )
>
> print(xyplot( -log10( pval ) ~ log2FoldChange,
+    resFly,
+    pch=20, cex=.2,
+    col=ifelse( resFly$padj<.1, "#FF000050", "#00000050" ),
+    axis = function( side, ... ) {
+       if( side=="bottom") {
+          panel.axis( side, outside=TRUE, at=seq(-14,14,by=1), labels=FALSE )
+          panel.axis( side, outside=TRUE, at=seq(-10,10,by=5), labels=TRUE )
+       }
+       if( side=="left") {
+          panel.axis( side, outside=TRUE, at=seq(0,25,by=1), labels=FALSE )
+          panel.axis( side, outside=TRUE, at=seq(0,25,by=5),
+             labels = do.call( expression,
+                lapply( seq(0,25,by=5), function(a)
+                   substitute( 10^-b, list(b=a) ) ) ) )
+       } },
+    xlab = "log2 fold change", ylab = "p value",
+    scales = list(
+       x = list( limits=c( -6, 6 ) ),
+       y = list( limits=c( 0, 25 ) ) ) ))
```

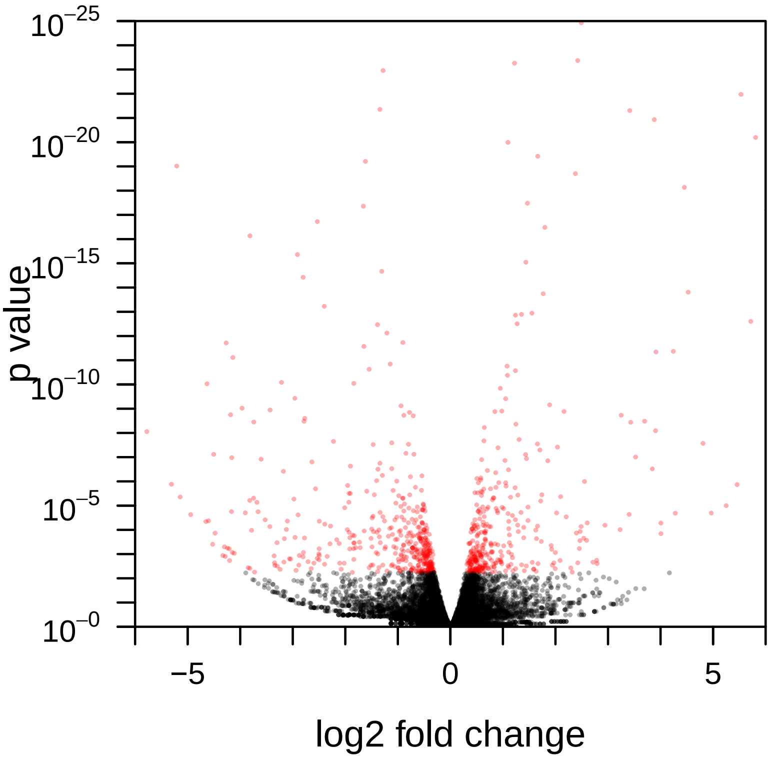

4.5 Hit density

As edgeR removes rows with zero counts, we should only look at those which are present in edgeR's result table:

```
> goodRows <- resFly$id %in% rownames(edgerResFlyDT$table)
```

We calculate the densities of hits as follows:

```
> densAll   <- density( log10(resFly$baseMean)[ goodRows ] )
> densHits  <- density( log10(resFly$baseMean)[ goodRows & resFly$padj < .1 ] )
> densedgDT <- density( log10(resFly$baseMean)[ goodRows ][ edgerResFlyDTPadj < .1 ] )
> densedgDC <- density( log10(resFly$baseMean)[ goodRows ][ edgerResFlyDCPadj < .1 ] )
> densedgNT <- density( log10(resFly$baseMean)[ goodRows ][ edgerResFlyNTPadj < .1 ] )
> densedgNC <- density( log10(resFly$baseMean)[ goodRows ][ edgerResFlyNCPadj < .1 ] )
```

We plot these for densities as follows:

```
> # pdf( "hits_dens_fly_all.pdf", width=6, height=4 )
>
> plot( densAll$x, densAll$y / 7, type="l", ylim=c(0,.05),
```

```
+    xlab="log10 mean", ylab="density", xaxs="i", yaxs="i", col="gray" )
> lines( densHits$x, densHits$y * densHits$n / densAll$n,
+    col="red", lwd=2 )
> lines( densedgDT$x, densedgDT$y * densedgDT$n / densAll$n,
+    col="blue", lwd=2, lty="dashed" )
> lines( densedgDC$x, densedgDC$y * densedgDC$n / densAll$n,
+    col="lightblue", lwd=2, lty="dashed" )
> lines( densedgNT$x, densedgNT$y * densedgNT$n / densAll$n,
+    col="blue", lwd=2, lty="solid" )
> lines( densedgNC$x, densedgNC$y * densedgNC$n / densAll$n,
+    col="lightblue", lwd=2, lty="solid" )
> text( densAll$x[310]+.2, densAll$y[310]/7, "x7", col="gray" )
```

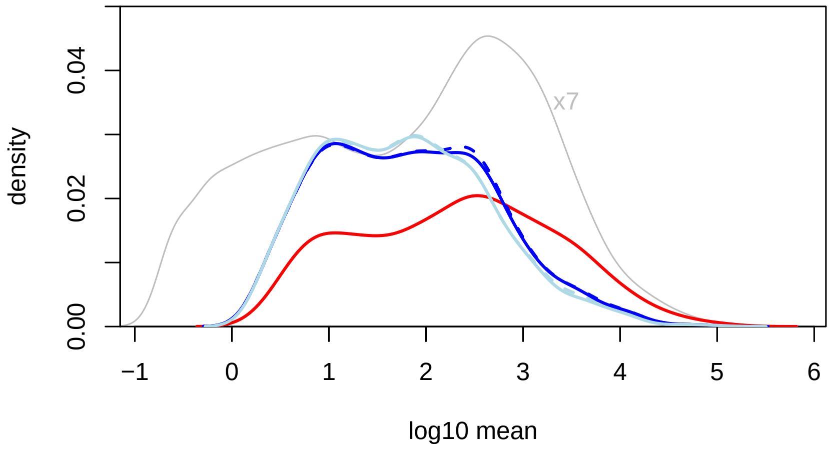

For this data set, there is hardly a noticeably difference between the two ways of specifying library size. Hence, we omitted the dashed lines from the figure in the paper:

```
> # pdf( "hits_dens_fly.pdf", width=6, height=4 )
>
> plot( densAll$x, densAll$y / 7, type="l", ylim=c(0,.05),
+    xlab="log10 mean", ylab="density", xaxs="i", yaxs="i", col="gray" )
> lines( densHits$x, densHits$y * densHits$n / densAll$n,
+    col="red", lwd=2 )
> lines( densedgNT$x, densedgNT$y * densedgNT$n / densAll$n,
+    col="blue", lwd=2, lty="solid" )
> lines( densedgNC$x, densedgNC$y * densedgNC$n / densAll$n,
+    col="lightblue", lwd=2, lty="solid" )
> text( densAll$x[310]+.2, densAll$y[310]/7, "x7", col="gray" )
```

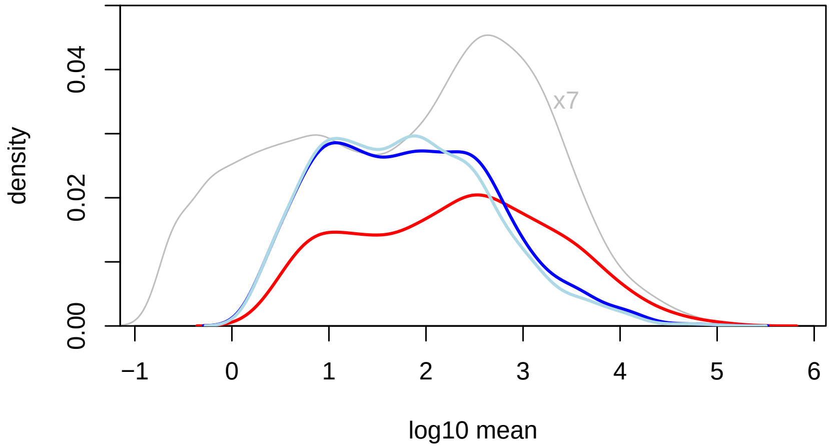

The effect of the tag-wise dispersion estimate (i.e., of moderated testing) is also hardly appreciable. This is fully expected because of the low number of replicates.

The paper gives numbers of hits, obtained as follows:

Percentage of genes with DESeq p value below 5%:

```
> length(which( resFly$pval < .05 )) / nrow( resFly )
```

```
[1] 0.1213860
```

Number of hits at 10% FDR, found by DESeq:

```
> length( which( resFly$padj < .1 ) )
```

```
[1] 864
```

The number of genes judged significant by edgeR is a bit higher and not very sensitive to the choice of library size specification and dispersion mode:

```
> c( DT = length( which( edgerResFlyDTPadj < .1 ) ),
+    DC = length( which( edgerResFlyDCPadj < .1 ) ),
+    NT = length( which( edgerResFlyNTPadj < .1 ) ),
+    NC = length( which( edgerResFlyNCPadj < .1 ) ) )
```

```
  DT   DC   NT   NC
1127 1104 1116 1097
```

The numbers of hits shared between DESeq and edgeR are as follows:

```
> list(
+   table( DESeq = resFly$padj[goodRows] < .1, edgeR_DT = edgerResFlyDTPadj < .1 ),
+   table( DESeq = resFly$padj[goodRows] < .1, edgeR_DC = edgerResFlyDCPadj < .1 ),
+   table( DESeq = resFly$padj[goodRows] < .1, edgeR_NT = edgerResFlyNTPadj < .1 ),
+   table( DESeq = resFly$padj[goodRows] < .1, edgeR_NC = edgerResFlyDCPadj < .1 ) )
```

```
[[1]]
       edgeR_DT
DESeq   FALSE  TRUE
  FALSE 13757   410
  TRUE    147   717

[[2]]
       edgeR_DC
DESeq   FALSE  TRUE
  FALSE 13772   395
  TRUE    155   709

[[3]]
       edgeR_NT
DESeq   FALSE  TRUE
  FALSE 13756   411
  TRUE    159   705

[[4]]
       edgeR_NC
DESeq   FALSE  TRUE
  FALSE 13772   395
  TRUE    155   709
```

4.6 Same-vs-same comparison

For the same-vs-same comparison of the two "A" samples, we first remove those genes which have zero counts in both samples and make a new CountDataSet, then run the standard analysis:[2]

```
> a <- counts(cdsFly)[,conditions(cdsFly)=="A"]
> cdsA <- newCountDataSet( a[rowSums(a)>0,], c( "A1", "A2" ) )
> cdsA <- estimateSizeFactors( cdsA )
> cdsA <- estimateVarianceFunctions( cdsA, method="blind" )
> resA <- nbinomTest( cdsA, "A1", "A2" )
```

We do the same with edgeR. (As edgeR does not have an option for "blind" variance estimation as DESeq does, we tell it first that both samples are replicates, then estimate the dispersion, and then change the group labels before the actual test:

```
> dglA <- DGEList( counts(cdsA), sizeFactors( cdsA ) * 1e7,
+    c( "A", "A" ) )
> dglA <- estimateCommonDisp( dglA )
> dglA <- estimateTagwiseDisp( dglA )

Using grid search to estimate tagwise dispersion.

> dglA$samples$group = c( "A1", "A2" )
> eResA <- exactTest( dglA, common.disp=FALSE  )
```

[2]With a DESeq version prior to 1.1.6, use "`pool=TRUE`" rather than "`method="blind"`"

```
Comparison of groups:  A2 - A1
```

Finally, we assume a Poisson model and fit a GLM:

```
> pvalPois <-
+    apply( counts(cdsA), 1, function(cl)
+       anova(
+          glm(
+             counts ~ sample,
+             data.frame( counts=cl, sample=colnames(counts(cdsA)) ),
+             family=poisson ),
+          test="Chisq" )$`P(>|Chi|)`[2] )
```

This code generates the ECDF curves (Fig. 2a):

```
> # pdf( file="repl_pval_fly_A_ecdf.pdf", width=6, height=6)
>
> print(ecdfplot( ~ data | which,
+    make.groups(
+       `DESeq, below 100` = resA$pval[ resA$baseMean < 100 ],
+       `DESeq, above 100` = resA$pval[ resA$baseMean > 100 ],
+       `DESeq, all` = resA$pval,
+       `edgeR, below 100` = eResA$table$p.value[
+           resA$baseMean < 100 ],
+       `edgeR, above 100` = eResA$table$p.value[
+           resA$baseMean > 100 ],
+       `edgeR, all` = eResA$table$p.value,
+       `Poisson, below 100` = pvalPois[ resA$baseMean < 100 ],
+       `Poisson, above 100` = pvalPois[ resA$baseMean > 100 ],
+       `Poisson, all` = pvalPois ),
+    layout=c(3,3), as.table=TRUE, xlab = "p value",
+    panel = function( ...) {
+       lsegments( 0, 0, 1, 1, col="darkgray" )
+       panel.ecdfplot( ... ) },
+    scales = list( tick.number = 4, cex=1 ) ))
```

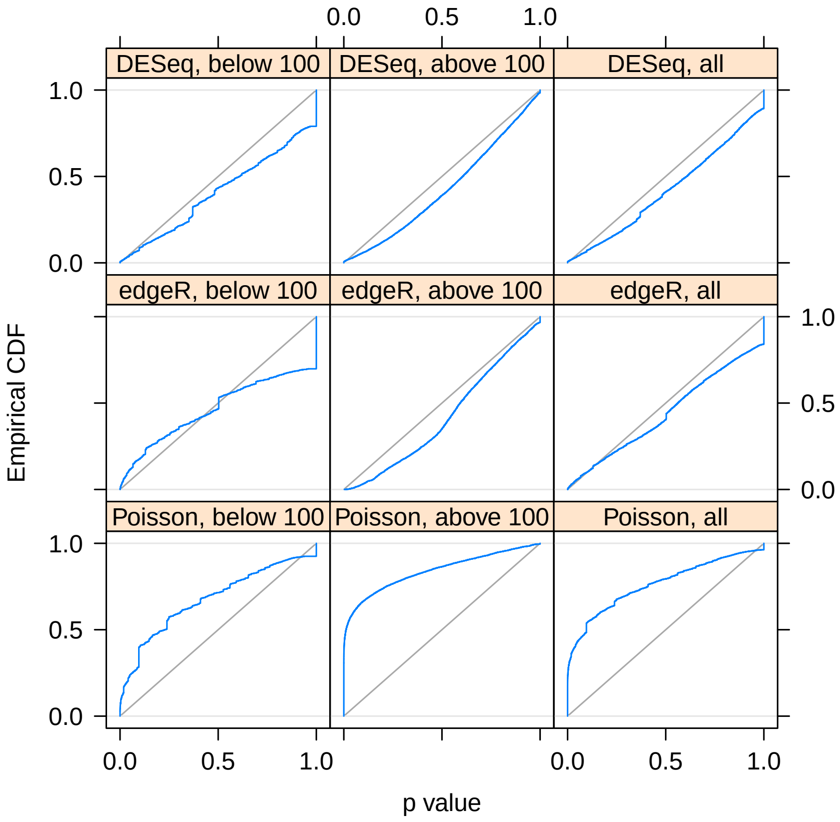

The zoom-in (Fig. 2b):

```
> # pdf( file="repl_pval_fly_A_ecdf_zoom.pdf", width=6, height=6)
>
> print(ecdfplot( ~ data | which,
+    make.groups(
+       `DESeq, below 100` = resA$pval[ resA$baseMean < 100 ],
+       `DESeq, above 100` = resA$pval[ resA$baseMean > 100 ],
+       `DESeq, all` = resA$pval,
+       `edgeR, below 100` = eResA$table$p.value[
+          resA$baseMean < 100 ],
+       `edgeR, above 100` = eResA$table$p.value[
+          resA$baseMean > 100 ],
+       `edgeR, all` = eResA$table$p.value,
+       `Poisson, below 100` = pvalPois[ resA$baseMean < 100 ],
+       `Poisson, above 100` = pvalPois[ resA$baseMean > 100 ],
+       `Poisson, all` = pvalPois ),
+    layout=c(3,3), as.table=TRUE, xlab = "p value",
+    panel = function( ...) {
+       lsegments( 0, 0, 1, 1, col="darkgray" )
+       panel.ecdfplot( ... ) },
```

```
+     xlim=c( -.005, .1 ), ylim=c( -.005, .1 ),
+     scales = list( tick.number = 4, cex=1 ) ))
```

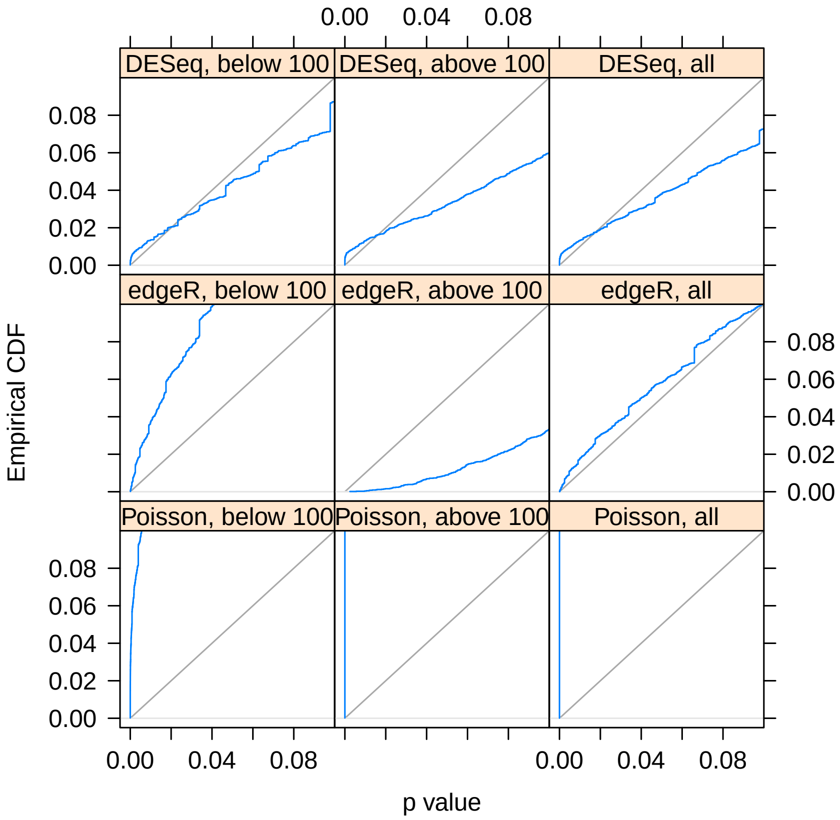

5 Analysis of neural stem cell data

We performed the same analysis as described in the previous section for the neural stem cell set. For completeness, we give here the full code to produce all the figures relating to this data set, but with only few comments, as the code is very similar to the already described code.

5.1 Analysis with DESeq and edgeR

As stated above and in the paper, we omit the first sample. Apart from that, the analysis is performed as before:

```
> cdsNS <- newCountDataSet( countsTableNS[,-1], condsNS[-1] )
> cdsNS <- estimateSizeFactors( cdsNS )
> cdsNS <- estimateVarianceFunctions( cdsNS )
> resNS <- nbinomTest( cdsNS, "GNS", "NS" )
```

For edgeR, we also have to explicitly remove the “GNSL” column. Apart from that, as before:

```
> colGNSL <- which( conditions(cdsNS) == "GNSL" )
> dglNS.D <- DGEList(
+    counts = counts(cdsNS)[,-colGNSL],
+    group = factor( conditions(cdsNS)[-colGNSL] ),
+    lib.size = 1e7*sizeFactors(cdsNS)[-colGNSL] )
> dglNS.D <- estimateCommonDisp( dglNS.D )
> dglNS.D <- estimateTagwiseDisp( dglNS.D )

Using grid search to estimate tagwise dispersion.

> edgerResNS.DT <- exactTest( dglNS.D, common.disp=FALSE )

Comparison of groups:  NS - GNS

> edgerResNS.DTPadj <- p.adjust( edgerResNS.DT$table$p.value, method="BH" )
> edgerResNS.DC <- exactTest( dglNS.D, common.disp=TRUE )

Comparison of groups:  NS - GNS

> edgerResNS.DCPadj <- p.adjust( edgerResNS.DC$table$p.value, method="BH" )
> libSizesNS <- c( G144=7604834, G166=13625570, G179=12291910,
+    CB541=12872125, CB660=10502656 )
> dglNS.N <- DGEList(
+    counts = counts(cdsNS)[,-colGNSL],
+    group = factor( conditions(cdsNS)[-colGNSL] ),
+    lib.size = libSizesNS[-colGNSL] )
> dglNS.N <- estimateCommonDisp( dglNS.N )
> dglNS.N <- estimateTagwiseDisp( dglNS.N )

Using grid search to estimate tagwise dispersion.

> edgerResNS.NT <- exactTest( dglNS.N, common.disp=FALSE )

Comparison of groups:  NS - GNS

> edgerResNS.NTPadj <- p.adjust( edgerResNS.NT$table$p.value, method="BH" )
> edgerResNS.NC <- exactTest( dglNS.N, common.disp=TRUE )

Comparison of groups:  NS - GNS

> edgerResNS.NCPadj <- p.adjust( edgerResNS.NC$table$p.value, method="BH" )
```

DESeq finds this number of significant hits at 10% FDR:

```
> length( which( resNS$padj < .1 ) )

[1] 673
```

For edgeR, the numbers are:

```
> c( DT = length( which( edgerResNS.DTPadj < .1 ) ),
+    DC = length( which( edgerResNS.DCPadj < .1 ) ),
+    NT = length( which( edgerResNS.NTPadj < .1 ) ),
+    NC = length( which( edgerResNS.NCPadj < .1 ) ) )

 DT  DC  NT  NC
316 525 256 452
```

5.2 Figures

Figure S4 has been made with this code:

```
> # pdf( "cv2_mean.pdf", width=4, height=4 )
>
> diagForGNS <- varianceFitDiagnostics( cdsNS, "GNS" )
> alpha <- dglNS.D$common.dispersion
> print(xyplot(
+    I( baseVar/baseMean^2 ) ~ baseMean,
+    diagForGNS[ diagForGNS$baseMean>0,],
+    panel = function( ... ) {
+       panel.hexbinplot( ... )
+       llines(
+          xg,
+          adjustScvForBias(
+            baseVarFunc(cdsNS,"GNS")( 10^xg ) / (10^xg)^2,
+            attr( rawVarFunc(cdsNS,"GNS")(NA), "size" ) ),
+          col="orange", lwd=2 )
+       llines( xg, ( 10^xg + alpha * (10^xg)^2 ) / (10^xg)^2,
+          col="orange", lwd=2, lty="dashed" )
+       for( sf in sizeFactors( cdsNS )[ conditions(cdsNS) == "GNS" ] )
+          llines( xg, 1 / (10^xg * sf), col="purple", lwd=1.5 ) },
+    trans = function(x) x^(1/4),
+    inv = function(x) x^4,
+    xbins = 80,
+    scales = list( x = list( log=TRUE, axs="i" ),
+       y = list( log=FALSE, axs="i", tick.number=8 ) ),
+    xlab = "mean", ylab="squared coefficient of variation"
+ ))
```

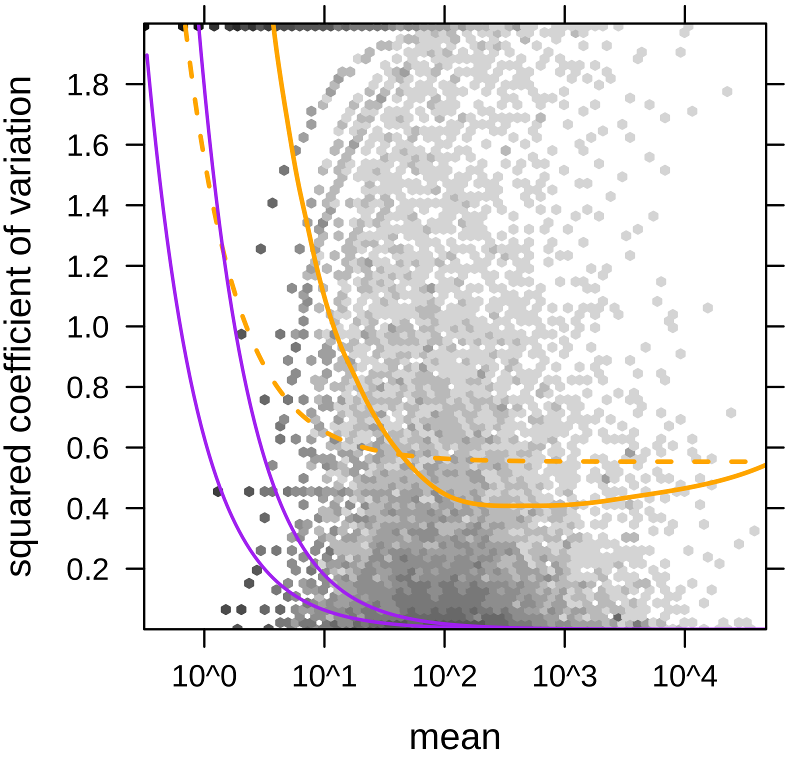

For Fig. S5, this code was used:

```
> # pdf( "DE.pdf", width=4, height=4 )
>
> print(xyplot(
+    log2FoldChange ~ I( baseMean ),
+    resNS,
+    pch=16, cex=.3,
+    col=ifelse( resNS$padj < .1, "#FF000040", "#00000040" ),
+    axis = function( side, ... ) {
+       if( side=="left") {
+          panel.axis( side, outside=TRUE, at=seq(-14,14,by=1), labels=FALSE )
+          panel.axis( side, outside=TRUE, at=seq(-10,10,by=5), labels=TRUE )
+       }
+       if( side=="bottom") {
+          panel.axis( side, outside=TRUE, at=seq(-2,10,by=1),
+             labels=paste( "10^", seq(-2,10,by=1), sep="" ) )
+       } },
+    xlab = "mean", ylab = "log2 fold change",
+    scales = list( x=list(log=TRUE), y=list(log=FALSE) ) ))
```

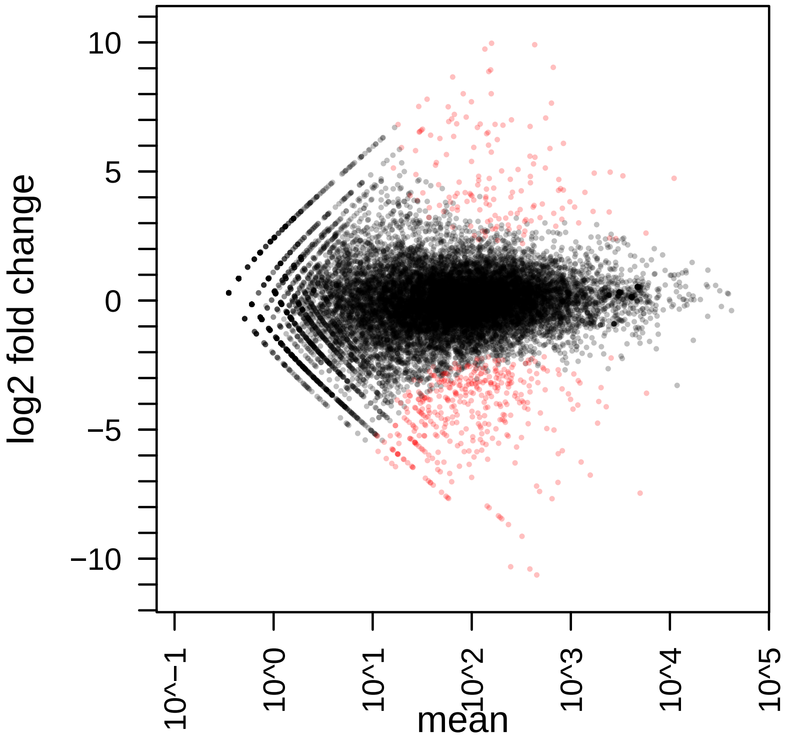

Figure S6, with the density estimates, was done with:

```
> # pdf( "hits_dens.pdf", width=6, height=4 )
>
> goodRowsNS <- resNS$id %in% rownames(edgerResNS.DT$table)
> densAll.NS   <- density( log10(resNS$baseMean)[ goodRowsNS ] )
> densHits.NS  <- density( log10(resNS$baseMean)[ goodRowsNS & resNS$padj < .1 ] )
> densedgDT.NS <- density( log10(resNS$baseMean)[ goodRowsNS ][ edgerResNS.DTPadj < .1 ] )
> densedgDC.NS <- density( log10(resNS$baseMean)[ goodRowsNS ][ edgerResNS.DCPadj < .1 ] )
> densedgNT.NS <- density( log10(resNS$baseMean)[ goodRowsNS ][ edgerResNS.NTPadj < .1 ] )
> densedgNC.NS <- density( log10(resNS$baseMean)[ goodRowsNS ][ edgerResNS.NCPadj < .1 ] )
> plot( densAll.NS$x, densAll.NS$y / 7, type="l", ylim=c(0,.07),
+    xlab="log10 mean", ylab="density", xaxs="i", yaxs="i",
+    col="gray" )
> lines( densHits.NS$x, densHits.NS$y * densHits.NS$n / densAll.NS$n,
+    col="red", lwd=2 )
> lines( densedgDT.NS$x, densedgDT.NS$y * densedgDT.NS$n / densAll.NS$n,
+    col="blue", lwd=2, lty="dashed" )
> lines( densedgDC.NS$x, densedgDC.NS$y * densedgDC.NS$n / densAll.NS$n,
+    col="lightblue", lwd=2, lty="dashed" )
> lines( densedgNT.NS$x, densedgNT.NS$y * densedgNT.NS$n / densAll.NS$n,
```

```
+    col="blue", lwd=2, lty="solid" )
> lines( densedgNC.NS$x, densedgNC.NS$y * densedgNC.NS$n / densAll.NS$n,
+    col="lightblue", lwd=2, lty="solid" )
> text( densAll.NS$x[310]+.2, densAll.NS$y[310]/7, "x7", col="gray" )
```

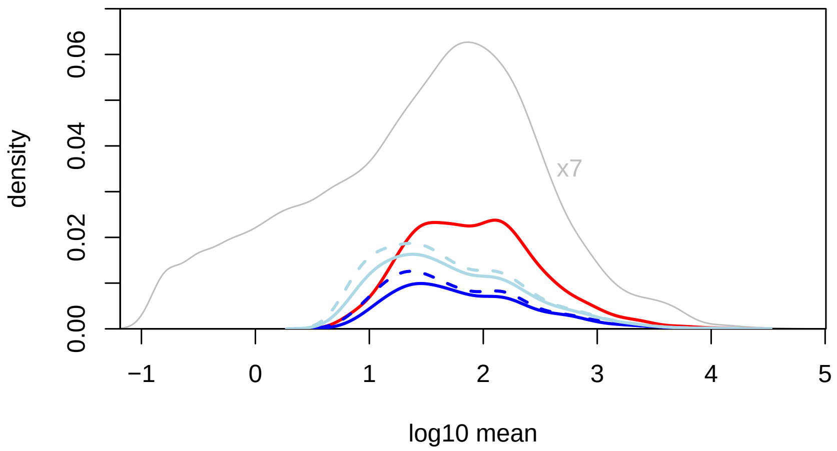

Finally, Figure S7 with the type-I error control histograms

```
> # pdf( file="repl_pval_A_ecdf.pdf", width=6, height=4.5)
>
> cdsGNS <- newCountDataSet( counts(cdsNS)[,1:2], c( "GNS_A", "GNS_B" ) )
> cdsGNS <- estimateSizeFactors( cdsGNS )
> cdsGNS <- estimateVarianceFunctions( cdsGNS, method="blind" )
> resGNS <- nbinomTest( cdsGNS, "GNS_A", "GNS_B" )
> medGNS <- median( getBaseMeansAndVariances( counts(cdsGNS),
+              sizeFactors(cdsGNS) )$baseMean )
> dglGNS <- DGEList( counts(cdsGNS), sizeFactors( cdsGNS ) * 1e7,
+    c( "GNS", "GNS" ) )
> dglGNS <- estimateCommonDisp( dglGNS )
> dglGNS <- estimateTagwiseDisp( dglGNS )

Using grid search to estimate tagwise dispersion.

> dglGNS$samples$group = c( "GNS_1", "GNS_2" )
> eResGNS <- exactTest( dglGNS, common.disp=FALSE )

Comparison of groups:  GNS_2 - GNS_1

> eMedGNS <- median( rowMeans(dglGNS$pseudo.alt) )
> print(ecdfplot( ~ data | which,
+    make.groups(
+       `DESeq lower half` = resGNS$pval[ resGNS$baseMean < medGNS ],
+       `DESeq upper half` = resGNS$pval[ resGNS$baseMean > medGNS ],
```

```
+        `DESeq all` = resGNS$pval,
+        `edgeR lower half` = eResGNS$table$p.value[
+           rowMeans(dglGNS$pseudo.alt) < eMedGNS ],
+        `edgeR upper half` = eResGNS$table$p.value[
+           rowMeans(dglGNS$pseudo.alt) > eMedGNS ],
+        `edgeR all` = eResGNS$table$p.value ),
+     layout=c(3,2), xlab = "p value", as.table=TRUE,
+     panel = function( ...) {
+        lsegments( 0, 0, 1, 1, col="darkgray" )
+        panel.ecdfplot( ... ) },
+     scales = list( tick.number=4, cex=1 ) ))
```

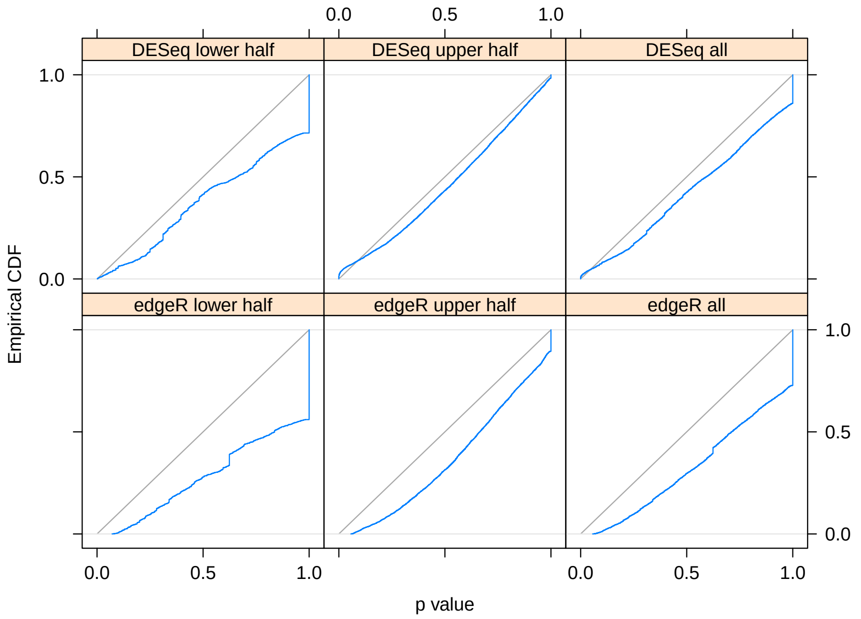

6 Working without replicates

To see how much hits we lose without replicates, we reduce the fly data set to only one sample per condition

```
> cdsFly2 <- cdsFly[ ,c( "A1", "B1" ) ]
> cdsFly2 <- estimateVarianceFunctions( cdsFly2, method="blind" )
> resFly2 <- nbinomTest( cdsFly2, "A", "B" )
```

Here we see a comparison of the hits with the full data set vs the reduced data set:

```
> addmargins( table( full = resFly$padj < .1, reduced = resFly2$padj < .1 ) )
```

```
       reduced
full    FALSE  TRUE   Sum
  FALSE 13546     2 13548
```

```
  TRUE     772    90   862
  Sum    14318    92 14410
```

The same for the neural stem cell data set:

```
> cdsNS2 <- cdsNS[ ,c( "G144", "CB541" ) ]
> cdsNS2 <- estimateVarianceFunctions( cdsNS2, method="blind" )
> resNS2 <- nbinomTest( cdsNS2, "GNS", "NS" )
> addmargins( table( full = resNS$padj < .1, reduced = resNS2$padj < .1 ) )

       reduced
full    FALSE  TRUE   Sum
  FALSE 15539    67 15606
  TRUE    410   202   612
  Sum   15949   269 16218
```

7 VST

To demonstrate the VST, we first make a new CountDataSet from the neural cell data, this time with all samples. To make clear that the dendrogram we get in the end was made without using class labels, we do not pass condition labels.

```
> # pdf( "heatmap.pdf" )
>
> cdsNS.all <- newCountDataSet( countsTableNS, rep( "dummy", ncol(countsTableNS) ) )
> cdsNS.all <- estimateSizeFactors( cdsNS.all )
> vsdNS <- getVarianceStabilizedData( cdsNS.all )
> distMatrix <- as.matrix(dist(t( vsdNS )))
> rownames( distMatrix ) <- c( "GNS (*)", "GNS (*)", "GNS", "GNS (L)", "NS", "NS" )
> heatmap.2( distMatrix, density.info="none", trace="none", revC=TRUE,
+    col=colorRampPalette(c("darkblue","yellow"))(100) )
```

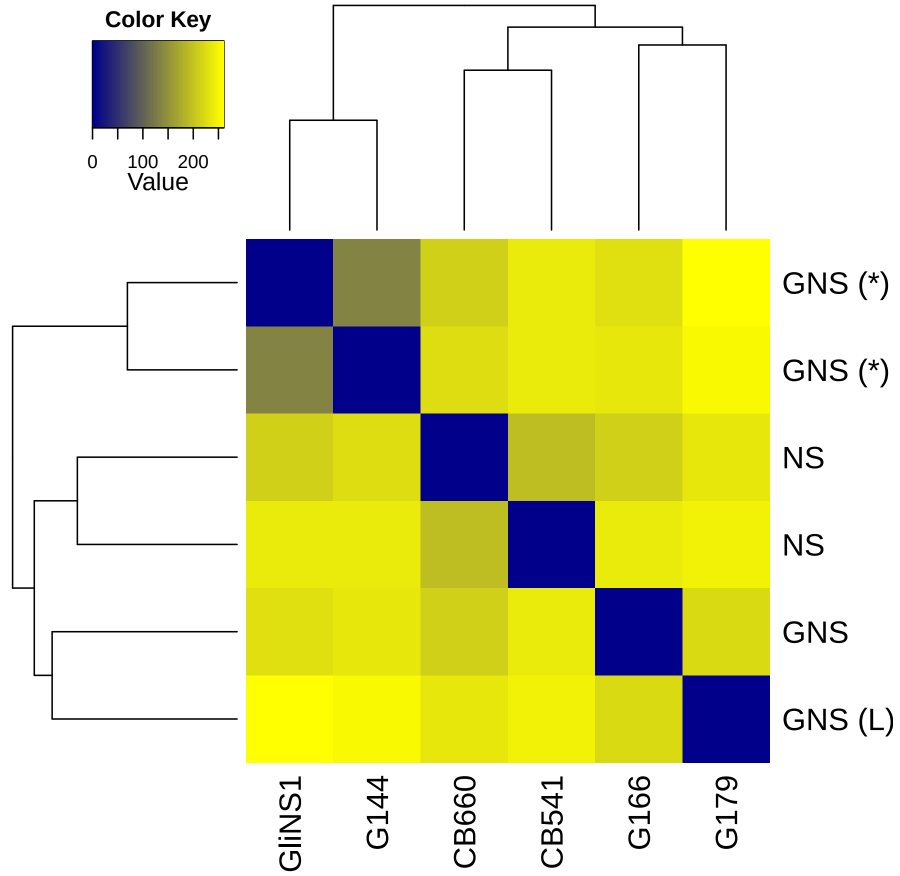

8 Yeast RNA-Seq data

For the yeast data, we have replicates, but no conditions to compare. DESeq's CountDataSet class is not designed to deal with this situation, and we fall back to the set of DESeq functions provided to directly work with matrix data:

```
> sfYeast <- estimateSizeFactorsForMatrix( countsYeast )
> # A function to get a base variance function:
> get_bvf <- function( cl ) {
+    rvf <- estimateVarianceFunctionForMatrix( countsYeast[,cl], sfYeast[cl] )
+    xim <- sum(1/sfYeast[cl])/length(cl)
+    function( q ) rvf( q ) + xim * q
+ }
> # The variance estimated between the replicates:
> bvf_dT_tech <- get_bvf( c("dT_ori","dT_tech") )
> bvf_dT_bio  <- get_bvf( c("dT_ori","dT_bio" ) )
> bvf_RH_tech <- get_bvf( c("RH_ori","RH_tech") )
> bvf_RH_bio  <- get_bvf( c("RH_ori","RH_bio" ) )
> # The common-scale means
> meansYeast <- getBaseMeansAndVariances( countsYeast, sfYeast )$baseMean
```

The plot of Fig. 7 is produced with

```
> #pdf("naga.pdf", width=4, height=4)
>
> layout( cbind(1:2), heights = c( 1, 3 ) )
> par( cex=.75 )
> par(mai=c(0.2, 1, 0.5, 0.5))
> plot( NULL, main="", xaxs="i", yaxs="i", ylab="density",
+    xlim=c( -.3, 4 ), ylim=c( 0, 1200 ), xaxt="n" )
> hist( log10( meansYeast ), breaks=30, add=TRUE )
> par(mai=c(1, 1, 0.01, 0.5))
> plot( NULL, log="x", ylim=c(0, .65), xaxs="i", yaxs="i",
+    xlab="mean", ylab="squared coefficient of variation",
+    xlim=c( 10^-.3, 10^4 ) )
> xg <- 10^seq( 0, 4, length.out=1000 )
> rect( xg[-1000], 1/(xg[-1000]*max(sfYeast)),
+    xg[-1], 1/(xg[-1000]*min(sfYeast)), col="#FFD0FF", lty="blank" )
> lines( xg, bvf_dT_bio(xg)/xg^2, col="brown" )
> lines( xg, bvf_dT_tech(xg)/xg^2, col="darkblue" )
> lines( xg, bvf_RH_bio(xg)/xg^2, col="brown", lty="dashed" )
> lines( xg, bvf_RH_tech(xg)/xg^2, col="darkblue", lty="dashed" )
> lines( xg, 1/xg, col="#FFD0FF" )
```

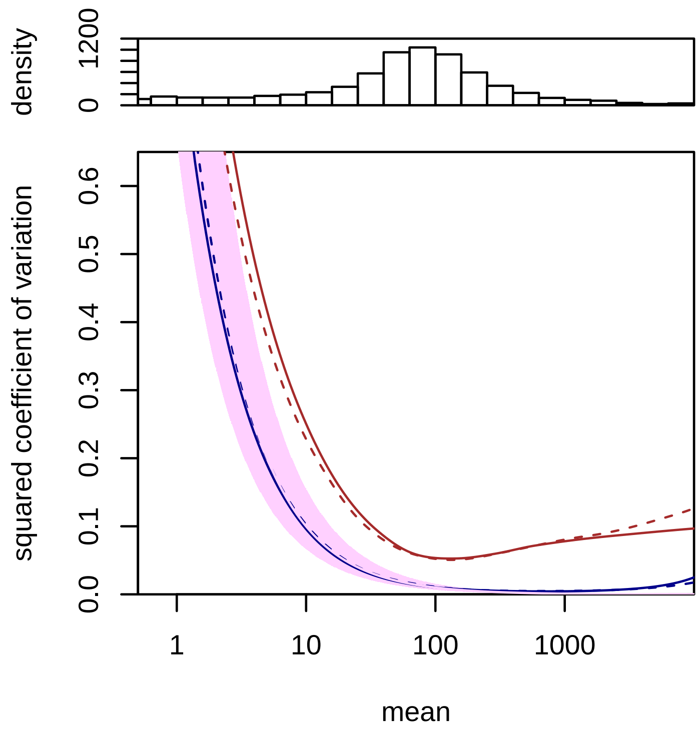

9 ChIP-Seq

We first look at a heat map for the Pol-II count data:

```
> cdsPolII.1 <- newCountDataSet( countsPolII, rep( "dummy", 12 ) )
> cdsPolII.1 <- estimateSizeFactors( cdsPolII.1 )
> heatmap( as.matrix( dist( t( getVarianceStabilizedData( cdsPolII.1 ) ) ) ),
+     cexCol=.7, cexRow=.7, symm=TRUE,
+     col=colorRampPalette(c("darkblue","yellow"))(100) )
```

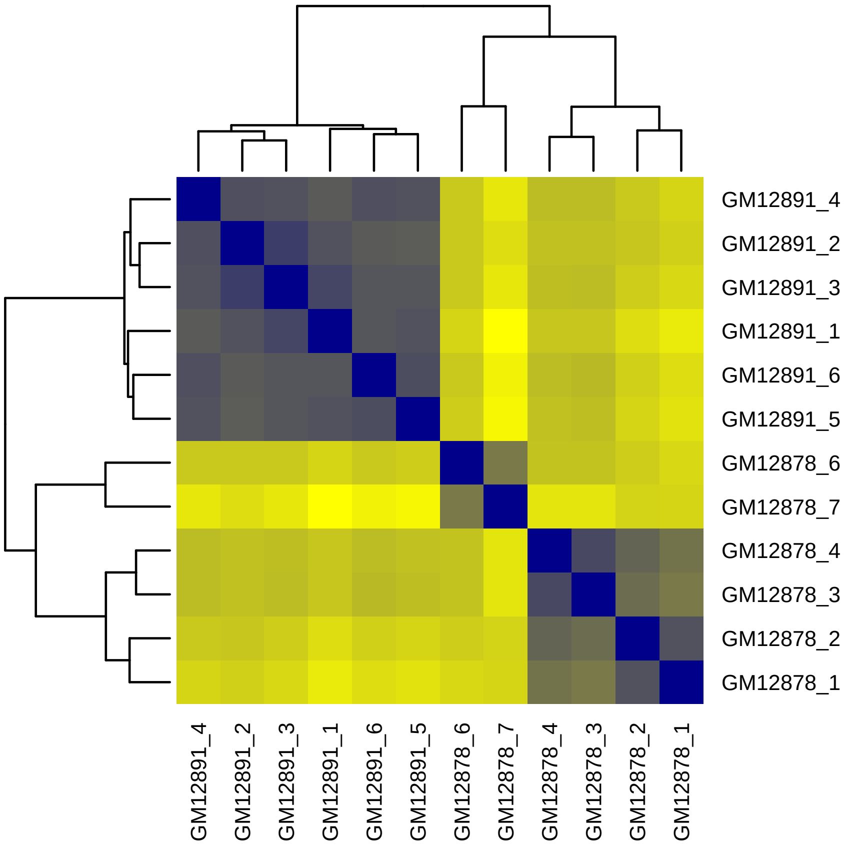

The heatmap shows clear evidence of batch effects for GM12878: Replicates 1 to 4 form one batch and 6 and 7 another one. For GM12891, replicates 1 to 3 form one batch and 4 to 6 another one.

For Fig. 6, we have to chose two replicates for each of the groups A1, A2, B1, and B2. In order to be balanced with respect to batches, we chose the replicates such that each batch is present in each group, as follows (Unused samples get denoted with an “x”):

```
> condsPolII <- c(
+    GM12878_1 = "A1", GM12878_2 = "A2", GM12878_3 = "x",
+    GM12878_4 = "x",  GM12878_6 = "A1", GM12878_7 = "A2",
+    GM12891_1 = "B1", GM12891_2 = "B2", GM12891_3 = "x",
+    GM12891_4 = "B1", GM12891_5 = "B2", GM12891_6 = "x" )
> cdsPolII.2 <- newCountDataSet( countsPolII, condsPolII )
> cdsPolII.2 <- estimateSizeFactors( cdsPolII.2 )
> cdsPolII.2 <- estimateVarianceFunctions( cdsPolII.2 )
> resPolII.A1vsA2 <- nbinomTest( cdsPolII.2, "A1", "A2" )
> resPolII.B1vsB2 <- nbinomTest( cdsPolII.2, "B1", "B2" )
> resPolII.A1vsB1 <- nbinomTest( cdsPolII.2, "A1", "B1" )
```

For the comparison of 4 replicates against 4, we use only the first letter:

```
> cdsPolII.3 <- newCountDataSet( countsPolII, substr( condsPolII, 1, 1 ) )
> conditions( cdsPolII.3 )
```

```
GM12878_1 GM12878_2 GM12878_3 GM12878_4 GM12878_6 GM12878_7 GM12891_1 GM12891_2
        A         A         x         x         A         A         B         B
GM12891_3 GM12891_4 GM12891_5 GM12891_6
        x         B         B         x
Levels: A B x
```

```
> cdsPolII.3 <- estimateSizeFactors( cdsPolII.3 )
> cdsPolII.3 <- estimateVarianceFunctions( cdsPolII.3 )
> resPolII.AvsB <- nbinomTest( cdsPolII.3, "A", "B" )
```

Number of hits at 10% FDR:

```
> table( resPolII.A1vsA2$padj < .1 )

FALSE
19028

> table( resPolII.B1vsB2$padj < .1 )

FALSE
19048

> table( resPolII.A1vsB1$padj < .1 )

FALSE  TRUE
14595  4460

> table( resPolII.AvsB$padj < .1 )

FALSE  TRUE
10619  8442
```

The same with a Poisson GLM:

```
> do_glm <- function( cds, cols )
+    apply( counts(cds)[, cols], 1, function(r)
+       anova(
+          glm( r ~ conditions(cds)[cols], family=poisson ),
+          test="Chisq" )$`P(>|Chi|)`[2] )
> pvalPois.A1vsA2 <- do_glm( cdsPolII.2, which( conditions(cdsPolII.2) %in% c("A1","A2") ) )
> pvalPois.B1vsB2 <- do_glm( cdsPolII.2, which( conditions(cdsPolII.2) %in% c("B1","B2") ) )
> pvalPois.A1vsB1 <- do_glm( cdsPolII.2, which( conditions(cdsPolII.2) %in% c("A1","B1") ) )
> pvalPois.AvsB   <- do_glm( cdsPolII.2, which( conditions(cdsPolII.3) %in% c("A","B") ) )
```

The ECDF plot of Fig. 6 is now made with:

```
> # pdf( "ecdf_Jan_pois.pdf", width = 6, height = 4.5 )
> print(ecdfplot( ~ data | which,
+    make.groups(
+       `D: A1 vs A2` = resPolII.A1vsA2$pval,
+       `D: B1 vs B2` = resPolII.B1vsB2$pval,
+       `D: A1 vs B1` = resPolII.A1vsB1$pval,
```

```
+        `D: A vs B`   = resPolII.AvsB$pval,
+        `P: A1 vs A2` = pvalPois.A1vsA2,
+        `P: B1 vs B2` = pvalPois.B1vsB2,
+        `P: A1 vs B1` = pvalPois.A1vsB1,
+        `P: A vs B`   = pvalPois.AvsB ),
+     layout=c(4,2), xlab = "p value", as.table=TRUE,
+     panel = function( ...) {
+        lsegments( 0, 0, 1, 1, col="darkgray" )
+        panel.ecdfplot( ... ) },
+     scales = list( tick.number=4, cex=1 ) ))
```

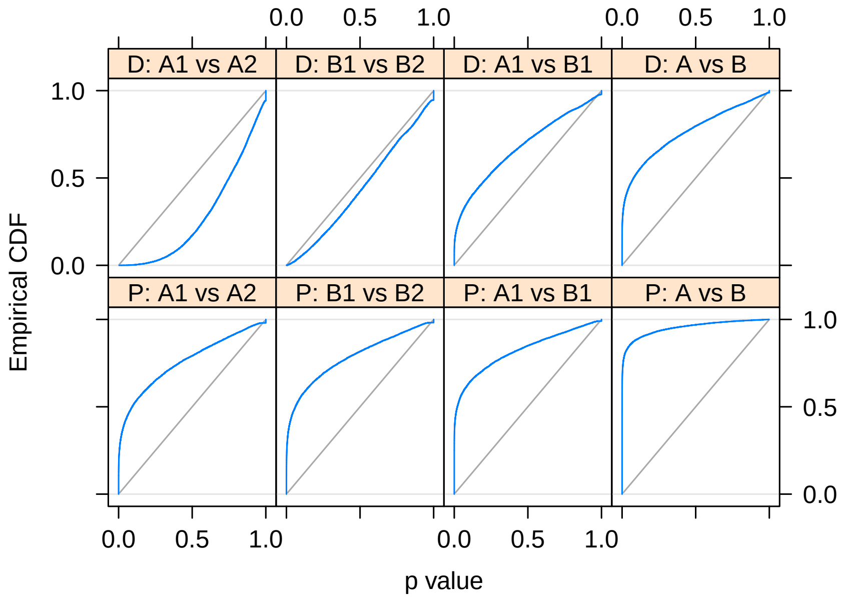

10 Simulation

The details of simulations in Supplementary Note G are given here.

10.1 Constant dispersion

The count data was drawn as follows

```
> # Number of genes to simulate
> ngenes <- 20000
> # True mean expression value across conditions drawn from an
> # exponential distribution
> true_q0middle <- rexp( ngenes, 1/250 )
> # These values might be typical size factors as could be found
> # in a real experiment
> sf <- c( .5, 1.7, 1.4, .9 )
```

```
> # Two replicates for each condition:
> conds <- c( "A", "A", "B", "B" )
> # Mark around 30% of the genes as differentially expressed:
> true_isDE <- runif( ngenes ) < .3
> # For the DE genes, draw a log fold change from a normal
> true_lfc <- rnorm( ngenes, sd=.7 ) * true_isDE
> # Go up and down from the true_q0middle according to these log
> # fold changes to get the values q for the conditions
> true_q0 <- data.frame(
+    A = true_q0middle * 2^(  true_lfc/2 ),
+    B = true_q0middle * 2^( -true_lfc/2 ) )
> # For the variance-mean relation, let's assume that the shape
> # assumed by edgeR is correct, i.e., variance = mean + alpha * mean^2
> # and use this alpha:
> alpha <- .015
> # Parametrise the NB to mean and variance:
> rnbinomMV <- function( n, mu, v )
+   rnbinom( n, prob = mu/v, size = mu^2/(v-mu) )
> # Now draw counts
> countsTable <-
+    sapply( 1:4, function(j)
+       sapply( true_q0[[ conds[j] ]], function(q)
+          rnbinomMV( 1, sf[j]*q, sf[j]*q + sf[j]^2 * q^2 * alpha ) ) )
> # The good rows:
> nonzero <- rowSums(countsTable)>0
```

Now, we analyse this count table:

```
> # A standard analysis with DESeq:
> cds <- newCountDataSet( countsTable, conds )
> cds <- estimateSizeFactors( cds )
> cds <- estimateVarianceFunctions( cds )
> res <- nbinomTest( cds, "A", "B" )
> # The same with edgeR. (edgeR gets the advantage of knowing the true
> # library sizes)
> dgl <- DGEList( counts=countsTable, group=conds, lib.size=sf*1e7 )
> dgl <- estimateCommonDisp(dgl)
> edgerRes <- exactTest( dgl )

Comparison of groups:  B - A

> edgerResPadj <- p.adjust( edgerRes$table$p.value, method="BH" )
```

Compare the results.

Type-I error control: Which percentage of genes without true DE have a p value below 5%? For DESeq:

```
> length(which( res$pval[nonzero]<.05 & !true_isDE[nonzero] )) / length(nonzero)

[1] 0.03345
```

For edgeR:

```
> length(which( edgerRes$table$p.value<.05 & !true_isDE[nonzero] )) / length(nonzero)
```

```
[1] 0.03295
```

Detection power: Which percentage of the true positives is detected at 10% FDR?
For DESeq:

```
> length(which( res$padj[nonzero]<.1 & true_isDE[nonzero] )) /
+    length(which( true_isDE[nonzero] ))
```

```
[1] 0.4376676
```

For edgeR:

```
> length(which( edgerResPadj<.1 & true_isDE[nonzero] )) /
+    length(which( true_isDE[nonzero] ))
```

```
[1] 0.4475536
```

FDR control:
For DESeq:

```
> length(which( res$padj[nonzero]<.1 & !true_isDE[nonzero] )) /
+    length(which( res$padj[nonzero]<.1 ))
```

```
[1] 0.06547406
```

For edgeR:

```
> length(which( edgerResPadj<.1 & !true_isDE[nonzero] )) /
+    length(which( edgerResPadj<.1 ))
```

```
[1] 0.06510326
```

For Fig. S10a, we used this code:

```
> print(ecdfplot( ~ data | which,
+    make.groups(
+       `DESeq` = res$pval[nonzero][ !true_isDE[nonzero] ],
+       `edgeR` = edgerRes$table$p.value[ !true_isDE[nonzero] ] ),
+    layout=c(2,1), as.table=TRUE, xlab = "p value",
+    panel = function( ...) {
+       lsegments( 0, 0, 1, 1, col="darkgray" )
+       panel.ecdfplot( ... ) },
+    xlim=c( -.005, .1 ), ylim=c( -.005, .1 ),
+    scales = list( tick.number = 4, cex=1 ) ))
```

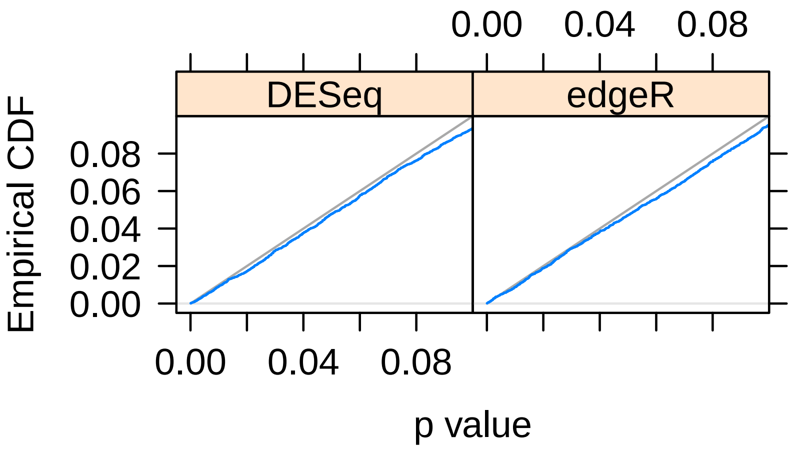

and for Fig. S10b

```
> # pdf( "pval_DE.pdf", width=4, height=4 )
>
> print(xyplot(
+    res$pval[true_isDE & nonzero] ~ edgerRes$table$p.value[true_isDE[nonzero]],
+    panel = function( ... ) {
+       panel.hexbinplot( ... )
+       llines( c(-35, 0), c(-35, 0), col="#30FF5090" ) },
+    trans = function(x) x^(1/4),
+    inv = function(x) x^4,
+    xbins = 150,
+    scales = list(
+       x = list(log=TRUE),
+       y = list(log=TRUE), apect=1 ),
+    xlim = c( 1e-15, 10 ),
+    ylim = c( 1e-15, 10 ),
+    aspect = 1,
+    xlab="p value from edgeR", ylab="p value from DESeq", asp=1
+ ))
```

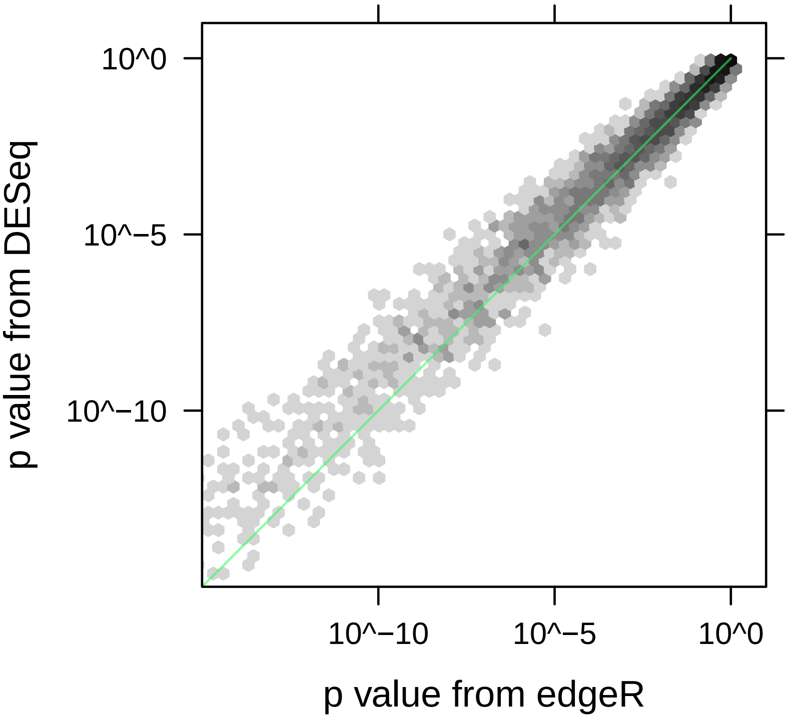

10.2 Non-constant dispersion

We prepare data as before:

```
> ngenes <- 20000
> true_q0middle <- rexp( ngenes, 1/250 )
> sf <- c( .5, 1.7, 1.4, .9 )
> conds <- c( "A", "A", "B", "B" )
> true_isDE <- runif( ngenes ) < .3
> true_lfc <- rnorm( ngenes, sd=.7 ) * true_isDE
> true_q0 <- data.frame(
+    A = true_q0middle * 2^(  true_lfc/2 ),
+    B = true_q0middle * 2^( -true_lfc/2 ) )
```

Now, we define a mean-dependent raw SCV value and use it in drawing the count data.

```
> alpha <- function(mu) .01 + 9/(mu+100)
> countsTable <-
+    sapply( 1:4, function(j)
+       sapply( true_q0[[ conds[j] ]], function(q)
+          rnbinomMV( 1, sf[j]*q, sf[j]*q + sf[j]^2 * q^2 * alpha( q ) ) ) )
```

Then, continue as before.

```
> nonzero <- rowSums(countsTable)>0
> cds <- newCountDataSet( countsTable, conds )
> cds <- estimateSizeFactors( cds )
> cds <- estimateVarianceFunctions( cds )
> res <- nbinomTest( cds, "A", "B" )
> dgl <- DGEList( counts=countsTable, group=conds, lib.size=sf*1e7 )
> dgl <- estimateCommonDisp(dgl)
> dgl <- estimateTagwiseDisp(dgl)
```

```
Using grid search to estimate tagwise dispersion.
```

```
> edgerRes <- exactTest( dgl, common.disp=FALSE )
```

```
Comparison of groups:  B - A
```

```
> edgerResPadj <- p.adjust( edgerRes$table$p.value, method="BH" )
```

Here is a plot showing the simulated (purple) and the fitted (solid red and green) raw SCV curves:

```
> scvPlot( cds )
> xg <- 10^seq( -2, 4, length.out=1000 )
> lines( xg, alpha( xg ), col="purple" )
```

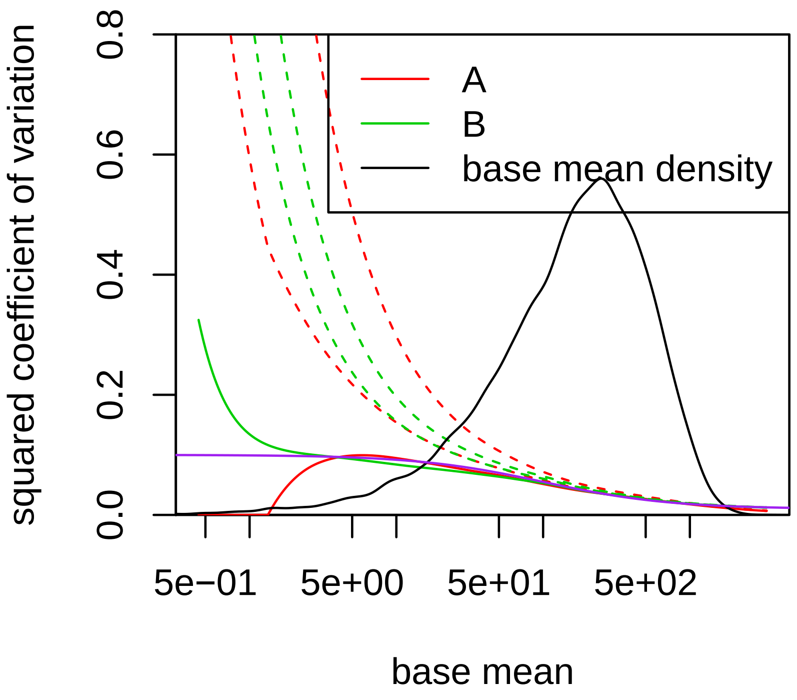

Finally, the ECDF plot

```
> # pdf( file="pvalcompsim2", width=8, height=6 )
>
> baseMeans <- t(t(countsTable)/sf)
> lowerHalf <- baseMeans < median( baseMeans )
> print(ecdfplot( ~ data | which,
+    make.groups(
+       `DESeq, below median` = res$pval[nonzero][
+          !true_isDE[nonzero] & lowerHalf[nonzero] ],
+       `DESeq, above median` = res$pval[nonzero][
+          !true_isDE[nonzero] & !lowerHalf[nonzero] ],
+       `DESeq, all`          = res$pval[nonzero][
+          !true_isDE[nonzero] ],
+       `edgeR, below median` = edgerRes$table$p.value[
+          !true_isDE[nonzero] & lowerHalf[nonzero] ],
+       `edgeR, above median` = edgerRes$table$p.value[
+          !true_isDE[nonzero] & !lowerHalf[nonzero] ],
+       `edgeR, all` = edgerRes$table$p.value[
+          !true_isDE[nonzero] ] ),
+    layout=c(3,2), as.table=TRUE, xlab = "p value",
+    panel = function( ...) {
+       lsegments( 0, 0, 1, 1, col="darkgray" )
+       panel.ecdfplot( ... ) },
+    xlim=c( -.005, .1 ), ylim=c( -.005, .1 ),
+    scales = list( tick.number = 4, cex=1 ) ))
```

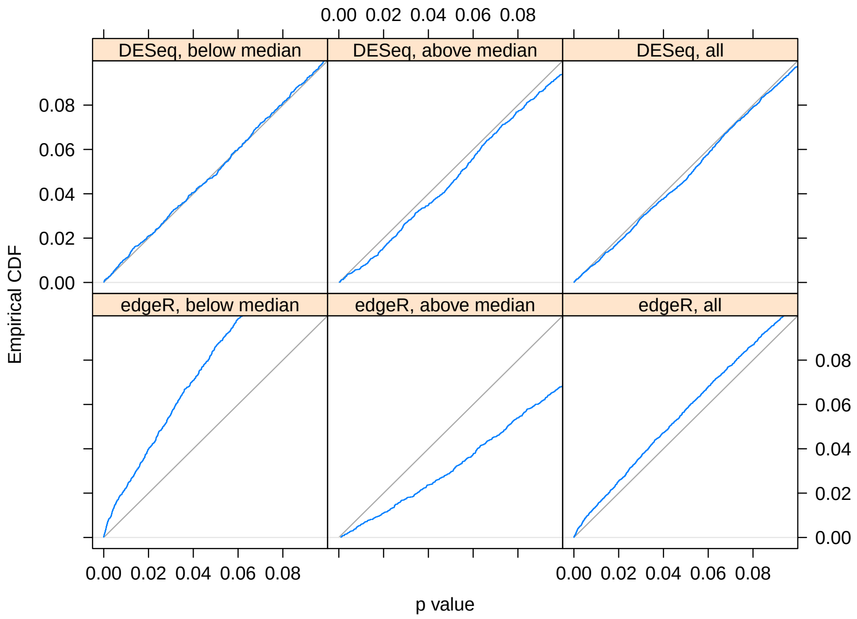

11 Other figures

11.1 Bias-adjustment function

The code for Fig. S1 is:

```
> # pdf("biasAdjust.pdf", width=4, height=4)
>
> plot( NULL, xlim=c(0,2), ylim=c(0,2),
+    xlab = expression( ( hat(sigma)^2 - hat(mu) ) / hat(mu)^2 ),
+    ylab = expression( gamma ) )
> xg <- seq( 0, 2, length.out=1000 )
> for( i in 2:15 )
+    lines( xg, adjustScvForBias( xg, i ),
+       col=colorRampPalette(c("red","black"))(15)[i] )
```

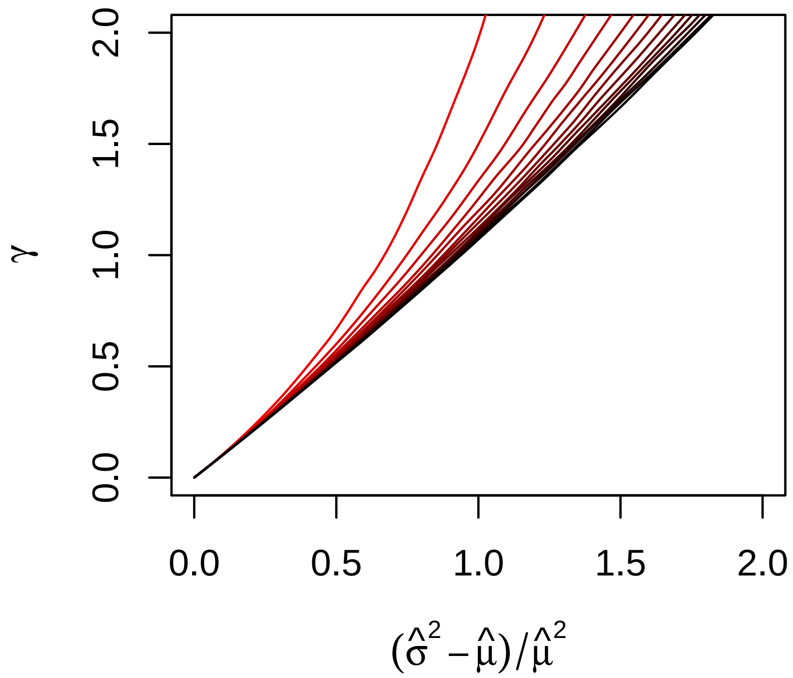

See the code of the functions *DESeq::adjustScvForBias* and *DESeq:::prepareScvBiasCorrectionFits* for details on how the variance adjustment is performed by means of fitting a LOESS regression through results from numerical simulations.

12 Figure of $p(a, b)$

The code for Fig. S2:

```
> # pdf("p1.pdf", width=4, height=4)
>
> dnbinomMV <- function( x, mu, v )
+   dnbinom( x, prob = mu/v, size = mu^2/(v-mu) )
> plot( 0:10000,
+    sapply( 0:10000, function(k)
+       dnbinomMV( k, 7000, 7000+.1*7000^2 ) *
+          dnbinomMV( 10000-k, 4000, 4000+.1*4000^2 ) ),
+    type='l', xlab="a", ylab="p(a,b)" )
> abline( v = 7000 / (7000+4000) * 10000 )
```

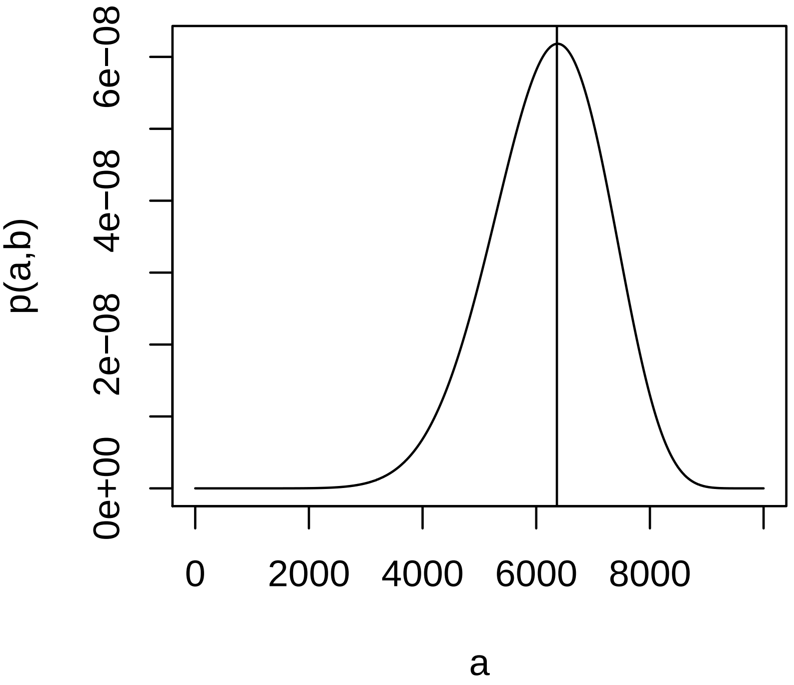

Finally, save all data:

```
> save.image( "suppl_ii.RData" )
```
